# Preparation and Utility of *N*-Alkynyl Azoles in Synthesis

**DOI:** 10.3390/molecules24030422

**Published:** 2019-01-24

**Authors:** Brandon Reinus, Sean M. Kerwin

**Affiliations:** 1 Department of Chemistry, University of Texas at Austin, Austin, TX 78712, USA; reinus@utexas.edu; 2 Department of Chemistry & Biochemistry, Texas State University, San Marcos, TX 78666, USA

**Keywords:** alkynes, cyclizations, natural products, carbenes, polymers

## Abstract

Heteroatom-substituted alkynes have attracted a significant amount of interest in the synthetic community due to the polarized nature of these alkynes and their utility in a wide range of reactions. One specific class of heteroatom-substituted alkynes combines this utility with the presence of an azole moiety. These *N*-alkynyl azoles have been known for nearly 50 years, but recently there has been a tremendous increase in the number of reports detailing the synthesis and utility of this class of compound. While much of the chemistry of *N*-alkynyl azoles mirrors that of the more extensively studied *N*-alkynyl amides (ynamides), there are notable exceptions. In addition, as azoles are extremely common in natural products and pharmaceuticals, these *N*-alkynyl azoles have high potential for accessing biologically important compounds. In this review, the literature reports of *N*-alkynyl azole synthesis, reactions, and uses have been assembled. Collectively, these reports demonstrate the growth in this area and the promise of exploiting *N*-alkynyl azoles in synthesis.

## 1. Introduction

**Figure 1:**
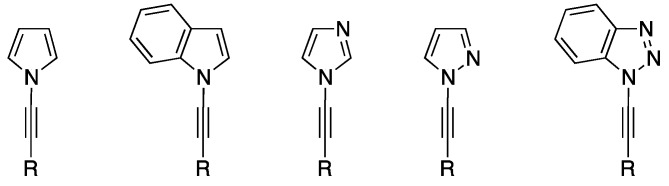
Examples of *N*-alkynyl azoles.

*N*-Alkynyl azoles, that is, alkynes containing an azole substituent through the ring nitrogen atom (Figure 1), have attracted a significant amount of interest over the last several decades, in part due to the significance of the two individual fragments: the azole and the alkyne. Alkynes are a fundamental building block and functional group that is indispensible to synthetic chemists, while azoles are extremely common in biomolecules and pharmaceuticals. The chemistry of *N*-alkynyl azoles represents just a fraction of the nitrogen-substituted alkyne field. Over the years, there have been many reviews of ynamines [1,2,3] and ynamides [3,4,5,6], and some of these have included *N*-alkynyl azoles within their content; however, the only review that primarily focused on this class of compounds was published in 2004 [7], and in the last 15 years there has been a significant amount of work published in this area.

Researchers were initially drawn to nitrogen-substituted alkynes for their structural relationship with enamines (nitrogen-subsituted alkenes); however, the general instability and highly reactive nature of ynamines made them undesirable research subjects and interest in them ultimately waned. Eventually, the discovery that electron-withdrawing groups on the nitrogen could stabilize these compounds led to a burgeoning interest in the synthesis and reactivity of ynamides and rejuvenated the field of nitrogen-substituted alkynes. Due to the diverse nature of the many different heterocycles that make up the azole family, there is not a single reactivity profile that defines all *N*-alkynyl azoles. However, in an analogous manner to ynamines being stabilized with electron-withdrawing groups, *N*-alkynyl azoles can be stabilized by modulating their electronic properties with functional group substitution. Over the past half century, since the first report of an *N*-alkynyl azole by Okamoto in 1970 [8], researchers have taken advantage of the properties of *N*-alkynyl azoles to develop new materials, new cytotoxic molecules, synthesize natural products, investigate new methodologies, and much more. Here we present a comprehensive review of the synthesis, reactions, and applications of *N*-alkynyl azoles, from Okamoto’s initial studies to the present.

## 2. Synthesis of *N*-Alkynyl Azoles

### 2.1. Elimination

**Scheme 1:**
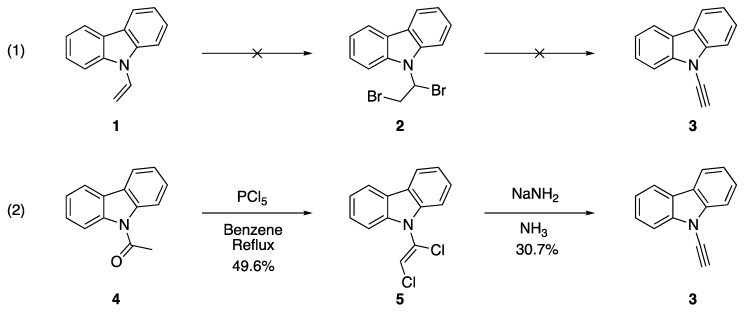
Kundu and Okamoto’s synthesis of *N*-ethynylcarbazole.

Dehydrohalogenation and other elimination procedures with *N*-alkenyl azoles constitute the original strategy towards preparing *N*-alkynyl azoles. These strategies are convenient for researchers due to the accessibility of the starting materials and the straightforward nature of the process. Dehydrohalogenation is a classic method for preparing alkynes and was an already established method for the synthesis of related *N*-alkynyl amines and amides [9,10]. The dehydrochlorination of *N*-(α,β-dichloroethenyl)carbazole (**5**) in 1970 by Kundu and Okamoto to form *N*-ethynylcarbazole (**3**) represents one of the earliest examples of this procedure applied to the synthesis of an *N*-alkynyl azole (Scheme 1) [8]. In their work, they found that they were unable to synthesize alkyne **3** from the α,β-dihalo derivative *N*-(α,β-dibromoethyl)carbazole (**2**) and sought an alternate route. They ultimately found success in generating **3** from *N*-acetylcarbazole (**4**) by treatment with phosphorus pentachloride in refluxing benzene followed by elimination with sodium amide in liquid ammonia.

**Scheme 2:**
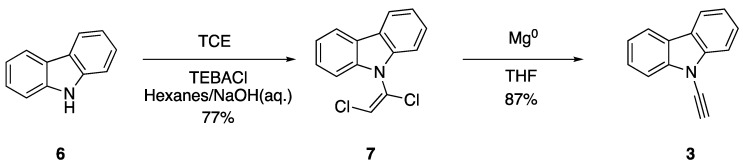
Pielichowski and Chrzaszcz’s phase-transfer synthesis of *N*-ethynylcarbazole.

In 1995, researchers Pielichowski and Chrzaszcz also became interested in the synthesis of *N*-ethynylcarbazole (**3**) and sought to improve on the original preparation by Kundu and Okomoto. The researchers found success in the synthesis of dichlorovinylcarbazoles via the reaction of carbazole and carbazole derivatives with trichloroethylene (TCE) under phase-transfer conditions, using triethylbenzylammonium chloride (TEBACl) as a phase-transfer catalyst (Scheme 2) [11]. This procedure allowed them to efficiently synthesize **7** from carbazole (**6**). Subsequent elimination with magnesium in dry tetrahydrofuran (THF) gave good yields of *N*-ethynylcarbazole (**3**). Pielichowski and Chrzaszcz highlighted the exceptional reactivity of *N*-ethynylcarbazole derivatives in their work, noting these compounds should be kept in the dark at low temperature and that they readily react with water in alcoholic solvents [11].

**Scheme 3:**
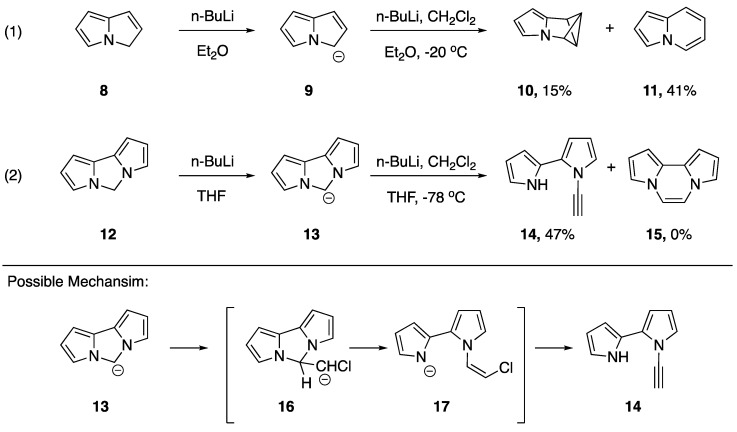
Burger and Dreier’s synthesis of an *N*-alkynyl pyrrole.

Burger and Dreier published a rather unusual synthesis of an *N*-alkynyl azole in 1983 [12]. Their synthesis, although unexpected, represents just the second report of a synthesis on an *N*-alkynyl azole and the first reported synthesis on an *N*-alkynyl pyrrole (Scheme 3). The researchers were studying the reactions of nitrogen containing heteroaromatic anions with chlorocarbene, an extension of previously established ring expansions of heterocyclic compounds with halocarbenes [13]. Some of the substrates the researchers studied exhibited the anticipated reactivity. For example, compound **9,** the lithium salt of 3*H*-pyrrolizine (**8**), reacted with chlorocarbene to give two compounds: the bicyclobutane derivative pyrrole-3-azabenzvalene **10** and indolizine (**11**), a ring-expanded product. On the other hand, they found that lithium anion **13**, formed by treating dipyrrolo [1.2c:2′,1′e]2*H*-imidazole (**12**) with *n*-butyllithium, reacted with chlorocarbene to give *N*-alkynyl pyrrole **14** in a 47% yield, while generating none of the expected ring expanded product **15**. Burger and Dreier proposed a mechanism for the formation of **14**, suggesting that an elimination of pyrrole from the initially formed **16**, followed by a dehydrohalogenation could generate the observed product [12].

**Scheme 4:**
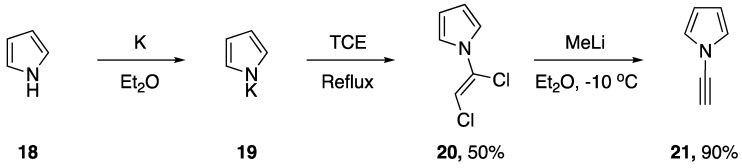
Paley and coworkers’ targeted synthesis of *N*-ethynylpyrrole.

In 1992, Paley and coworkers published the first targeted synthesis of an *N*-alkynyl pyrrole [14]. The researchers were interested in studying the properties and characteristics of poly(diacetylenes) and using computer modeling to aid in both the prediction of their properties and in the screening of potentially interesting target compounds. This work led them to develop a synthesis of *N*-ethynyl-pyrrole (**21**, Scheme 4). Paley’s strategy parallels the route of Kundu and Okamoto for the synthesis of *N*-ethynylcarbazole. Starting with pyrrole (**18**) they were able to easily generate the potassium salt **19**. Following decanting of the solvents the salt was refluxed in TCE overnight and *N*-(α,β-dichlorovinyl)pyrrole (**20**) was obtained in moderate yields. Subsequent elimination with methyllithium in diethyl ether gave the desired *N*-ethynyl pyrrole in a 90% yield.

In 1994, Trofimov also published a synthesis of *N*-ethynylpyrrole (**21**) [15]. Trofimov’s research program was focused on the chemistry of *N*-substituted pyrroles, and in an attempt to investigate the chemistry of *N*-ethynyl pyrrole, they found Paley and coworkers’ synthesis unsatisfactory. Trofimov was able to avoid the use of both potassium metal and TCE as a solvent by generating dichloroacetylene in situ. His procedure involves using potassium *tert*-butoxide in THF with TCE to generate the dichloroacetylene to which pyrrole readily adds. Trofimov also uses methyllithium for the elimination.

**Scheme 5:**
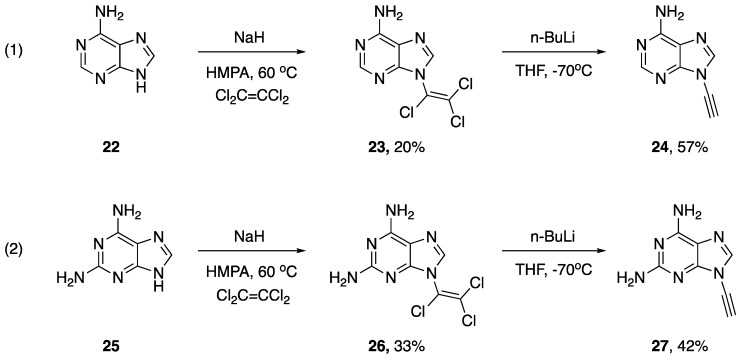
Zemlicka’s synthesis of *N*^9^-alkynyl purines.

As part of their work studying the biological activity of various chloro-enamines and *N*-alkynyl amines, in 1994, Zemlicka described a synthesis of *N*-alkynyl purines (Scheme 5) [16]. During their investigations, they found the reaction of adenine (**22**) with dichloroacetylene to be disappointing, providing the corresponding *N*^9^-dichloroenamine in only a 3% yield. Ultimately, they did have success with the addition of purines **22** and **25** to tetrachloroethylene using sodium hydride as a base and hexamethylphosphoramide (HMPA) as a solvent. The authors suggest these reactions likely proceed via an addition-elimination mechanism (since tetrachloroethylene is unable to react via elimination-addition). Subsequently, the researchers were able to convert the *N*^9^-(trichlorovinyl)purine derivatives **23** and **26** into the respective *N*^9^-alkynyl purines by carefully subjecting them to n-BuLi in THF at −70 °C, providing **24** and **27** in 57% and 42% yields, respectively. In addition, the authors mention that although they were able to synthesize *N*^9^-(dichlorovinyl)purines and *N*^9^-(chlorovinyl)purines, they only managed to eliminate the trichlorovinyl derivatives to the corresponding alkynes.

**Scheme 6:**
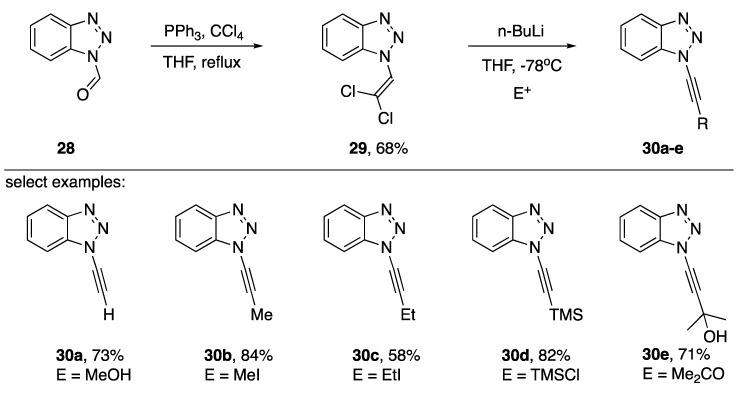
Katritzky’s ‘Corey-Fuchs’ approach to various N^1^-alkynyl benzotriazoles.

*N*^1^-alkynyl benzotriazoles have found applications in the synthesis of disubstituted alkynes and in an alternative procedure to the Arndt-Eistert homologation of carboxylic acids [17,18]. In 2006, Katritzky published a dehydrohalogenation strategy for the rapid synthesis of various *N*^1^-alkynyl benzotriazoles (Scheme 6) [19]. This approach is appealing both for the relatively few steps involved and the accessibility of the starting materials. Starting from commercially available **28**, treatment with carbon tetrachloride and triphenylphosphine in refluxing THF afforded the 1-(2,2-dichlorovinyl)-benzotriazole (**29**) in a 68% yield. Dehydrohalogenation of intermediate **29** followed by trapping of the transiently formed lithium acetylide with various electrophiles gave *N*^1^-alkynyl benzotriazoles **30a**–**e** in varying yields. Katritzky observed a significant drop in yield when changing the electrophile from methyl iodide to ethyl iodide suggesting this strategy might not be suitable for synthesizing *N*^1^-alkynyl benzotriazoles with branched alkyl substitutions on the alkyne.

**Scheme 7:**
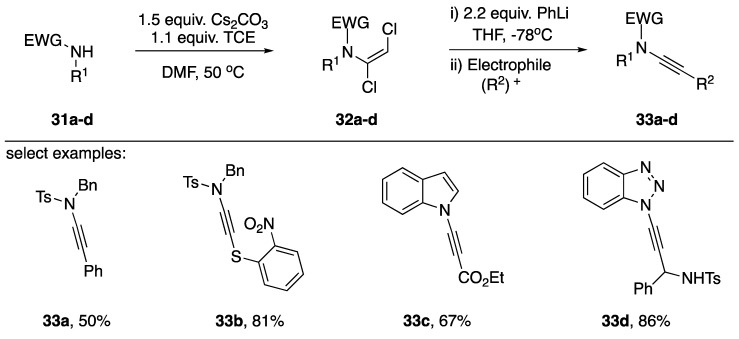
Anderson’s synthesis of *N*-alkynyl amides and *N*-alkynyl azoles.

In 2015, Anderson published a more general approach to *N*-alkynyl amide synthesis, including several examples of *N*-alkynyl azoles (Scheme 7) [20]. 

In a similar approach as Katritzky, the researchers found they were able to trap an intermediate *N*-alkynyl lithium acetylide with various electrophiles. Interestingly, after transmetalation with ZnCl_2_ the researchers were able to perform an in situ Negishi coupling with iodobenzene to afford the *N*-alkynyl tosylamide **33a**. Additionally, the researchers managed to generate sulfur-substituted *N*-alkynyl tosylamides when using the sulfur based electrophile **33b**. Although Anderson reported the synthesis of many 1,2-vinyldichlorides of azoles, there were only two examples presented in which they completed the synthesis to generate the *N*-alkynyl azole. Using their strategy, they were able to make *N*-alkynyl indole **33c** in a 67% yield and *N*^1^-alkynyl benzotriazole **33d** in an 86% yield. The author suggests the advantages of this approach consist of a larger substrate tolerance and greater practicality to metal-catalyzed strategies; however, the use of phenyllithium precludes the use of substrates with electrophilic functional groups.

**Scheme 8:**
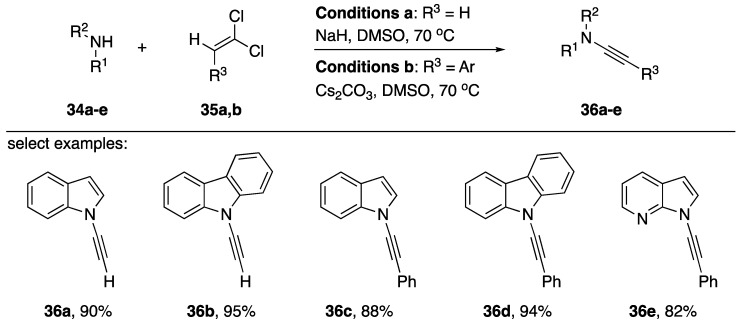
Zhao’s transition-metal free one-step synthesis of *N*-alkynyl amides.

Zhao reported a transition-metal free one-step synthesis of *N*-alkynyl amides in 2018 (Scheme 8) [21]. In the publication, the researchers included several examples of the application of the new methodology towards the synthesis of *N*-alkynyl azoles. They synthesized *N*-alkynyl indoles **36a**, **36c**, and **36e** in 90%, 88%, and 82% yields, respectively. Additionally, *N*-alkynyl carbazoles **36b** and **36d** were isolated in 95% and 94% yields. The methodology does have limitations: Zhao mentions their failed attempts to prepare alkyl substituted alkynes. Zhao suggests the reaction proceeds first by an elimination of the 1,1-divinylchlorides to the corresponding alkynyl-chloride followed by an addition of the nitrogen nucleophile and subsequent elimination to the *N*-alkynyl amide or *N*-alkynyl azole. In the case of aromatic substrates, the researchers explain that the nucleophile may prefer to add α to the chloride because the aromatic ring can stabilize the negatively charged intermediate. However, in the case of alkyl substrates this stabilization is not available and chloroenamides are isolated, suggesting a β-position addition of the nucleophiles is preferred in these cases.

**Scheme 9:**
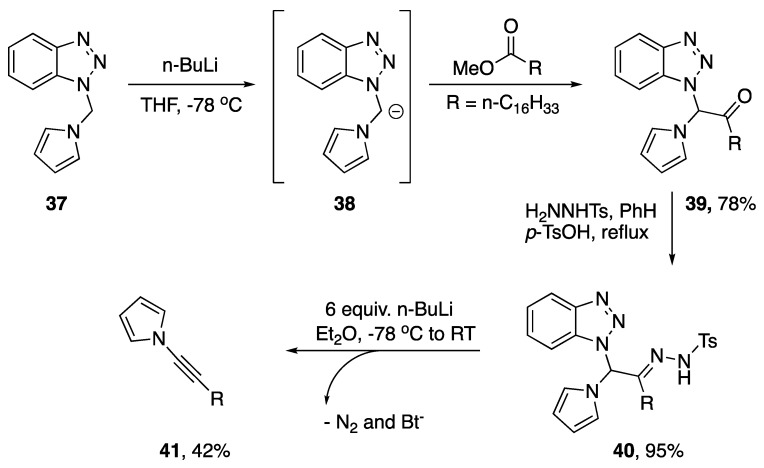
Katritzky’s Shapiro-type synthesis on an *N*-alkynyl pyrrole.

In addition to dehydrohalogenation methods for the synthesis of *N*-alkynyl azoles there have been several publications reporting eliminations with alternative leaving groups. Interestingly, nearly all of these methods have been disclosed by Katritzky. In 1997, Katritzky reported a Shapiro-type elimination as a new method for synthesizing alkynes from esters and within this publication the author discloses the synthesis of an *N*-alkynyl pyrrole (Scheme 9) [22]. Starting with pyrrole-benzotriazole intermediate **37**, the researchers were able to deprotonate and subsequently acylate the bridging methylene generating ketone **39** in good yields. Reaction of the ketone with (*p*-toluenesulfonyl)hydrazine easily converted the ketone into the corresponding hydrazone **40**, and by subjecting hydrazone **40** to six equivalents of *n*-BuLi the authors were able to synthesize *N*-alkynyl pyrrole **41** in a moderate 42% yield.

**Scheme 10:**
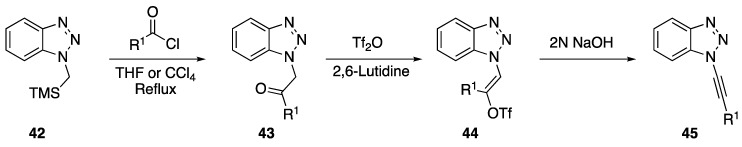
Katritzky’s *N*^1^-alkynyl benzotriazole synthesis.

In 2001 and 2002, Katritzky reported on two separate transformations involving *N*-alkynyl benzotriazoles. In 2001, Katritzky developed an alternative protocol to the Arndt-Eistert homologation that involves an *N*^1^-alkynyl benzotriazole intermediate [17]; and in 2002, the researchers used *N*^1^-alkynyl benzotriazoles in the synthesis of disubstituted alkynes [18]. In both these publications, Katritzky used the same method to prepare the *N*^1^-alkynyl benzotriazoles (Scheme 10). Starting with (chloromethyl)trimethylsilane and benzotriazole they easily prepared the starting benzotriazole **42**. This, upon treatment with an acid chloride in refluxing solvent, smoothly afforded ketones such as **43**. Subsequent reaction of the ketones with triflic anhydride gave the related enol-triflates **44**. Finally, elimination with aqueous NaOH gave *N*^1^-alkynyl benzotriazoles **45**. The elimination of enol-phosphates has also been applied towards the synthesis of *N*-alkynyl azoles. In 2015, An and Dong published a one-pot enol-phosphate-elimination strategy towards the synthesis of various *N*-alkynyl benzimidazoles and *P*-alkynyl phosphonates [23].

### 2.2. Alkynyliodonium Salts

**Scheme 11:**
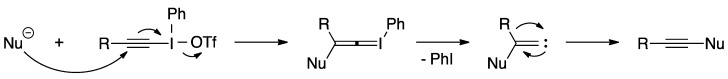
Mechanism of alkynyliodonium salts as alkyne-transfer reagents.

Alkynyliodonium salts have received a lot of attention from the synthetic community for their usefulness as synthons for cationic alkynes and have been used extensively as alkyne-transfer reagents [24,25]. It is widely believed that these reagents undergo a Michael-type addition followed by a rearrangement to transfer an alkyne to a nucleophile [25]. This process involves three steps: (1) the nucleophile attacks the β-carbon of the alkynyliodonium salt generating an alkenylideneiodonium ylide, (2) the ylide eliminates iodobenzene and generates an alkenylidenecarbene, and (3) the carbene rearranges into an alkyne (Scheme 11).

**Scheme 12:**
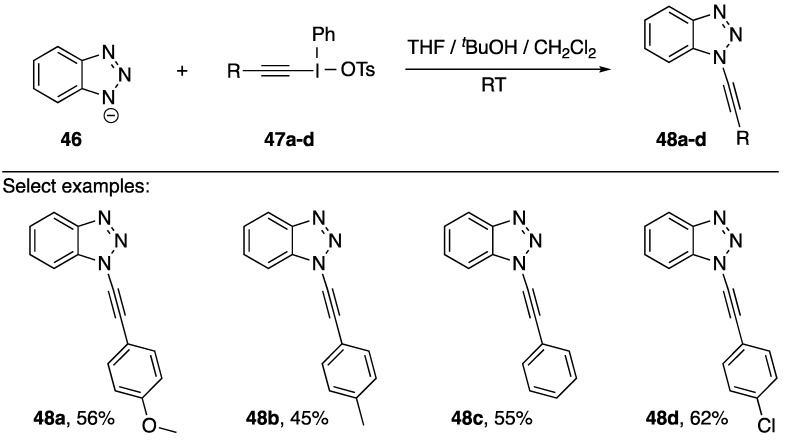
Kitamura’s synthesis of N^1^-alkynyl benzotriazoles.

In 1998, Kitamura reported using alkynyl(phenyl)iodonium tosylates as alkyne transfer reagents for the synthesis of *N*^1^-alkynyl benzotriazoles (Scheme 12) [26,27]. They explored a handful of different transfer reagents (**47a**–**d**) and found that they could form the corresponding alkynes **48a**–**d** in moderate yields (45–62%). They were able to provide support for the mechanism by trapping the intermediate carbene with *^t^*BuOH and also trapping the carbene via 1,5 C-H insertion. However, this methodology is limited by the mechanism, Kitamura suggests that the substituent on the alkynylation reagent needs to have a high migratory aptitude and that it cannot be aliphatic.

**Scheme 13:**
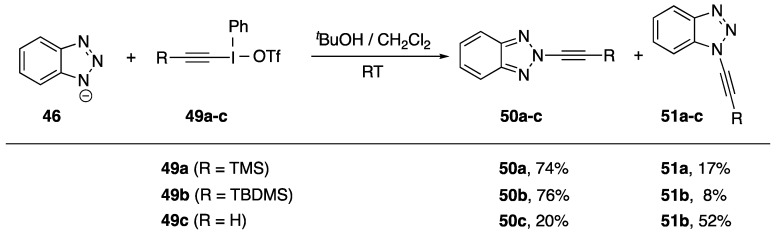
Kitamura’s synthesis of N^2^-alkynyl benzotriazoles.

More than a decade after their initial publication, Kitamura et al. published a follow-up synthesis of *N*^2^-alkynyl benzotriazoles (Scheme 13) [28]. Kitamura believes this is the first example of a regioselective functionalization of benzotriazole at the 2-position. The researchers found that when carrying out an alkyne transfer reaction with trimethylsilylethynyliodonium triflate (**49a**) under very similar reaction conditions to their previously published work, they isolated the isomeric *N*^2^-alkynyl benzotriazole **50a** as the major product of the reaction. Alkyne transfer reagent **49b** performed similarly; however, reagent **49c** in which the alkyne has no substitution gave a near statistical ratio of the two isomers. Kitamura suggests that the differences in regioselectivity may be attributable to a steric interaction, but he does not provide any insight into the origin of the regioselective preference for the alkyne-transfer reactions with arylethynyliodonium salts.

More recently, Davydov and co-workers studied the *N*-alkynylation of benzotriazole with alkynyliodonium reagent **47c** (R = Ph) under palladium catalysis [29]. These authors report selectivity for *N*^1^- versus *N*^2^-alkynylation ranging from 3:1 up to 21:1, with the highest regioselectivity for *N*^1^-alkynylation obtained using catalytic alumina-supported Pd in acetonitrile. Huang and Zhu have reported using a polymer-supported alkynyliodonium reagent to prepare *N*^1^-alkynylbenzotriazole **48c** in 60% yield [30].

**Scheme 14:**
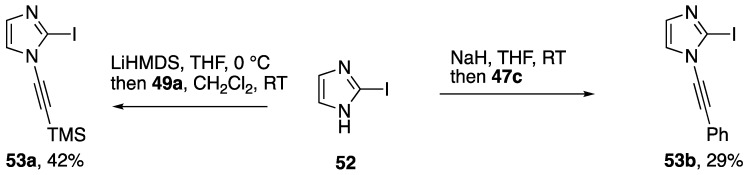
Kerwin’s synthesis of *N*-alkynylimidazoles using alkynyliodonium reagents.

**Scheme 15:**
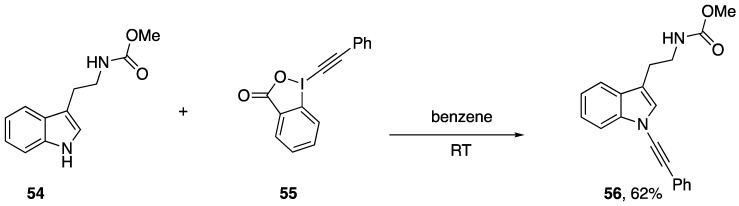
Bisai’s synthesis of an *N*-alkynylindole.

Alkynyliodonium and alkynylbenziodoxolone reagents have also been employed in alkynyl transfer to other heterocycles. Kerwin demonstrated that the iodonium reagents **47c** and **49a** can be used to access the *N*-alkynylimidazoles **53a,b** (Scheme 14) by reaction with the anion of 2-iodoimidazole (**52**), albeit in modest yields [31,32]. As part of a study on the oxidative alkynylation of 2-oxindoles, Bisai and co-workers prepared the *N*-alkynylindole **56** using the ethynylbeziodoxolone **55** (Scheme 15) [33].

**Scheme 16:**
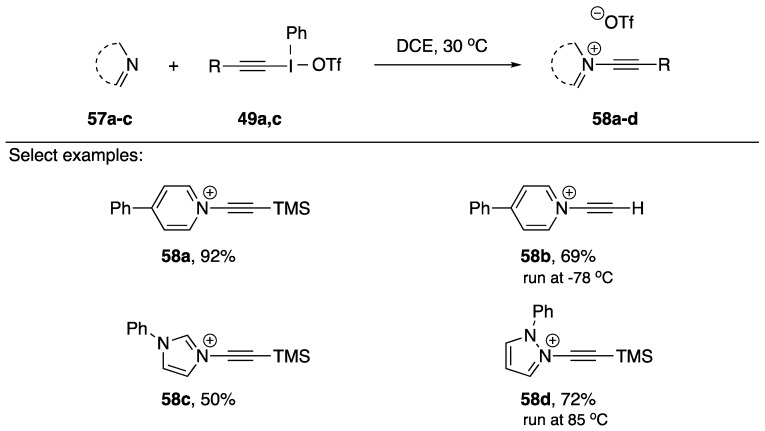
Toriumi and Uchiyama’s synthesis of *N*-alkynyl azolium salts.

In 2018, Toriumi and Uchiyama published a synthesis of *N*-akynyl pyridinium salts as part of a research program investigating cationic nitrogen-embedded polycyclic aromatic hydrocarbons (cNe-PAHs) (Scheme 16) [34]. cNe-PAHs have many potential applications [35], but are challenging to synthesize. The researchers postulated that *N*-alkynyl pyridinium salts could serve as useful precursors for the synthesis of cNe-PAHs, and in the process of conducting their research they also synthesized two *N*-alkynyl azolium salts. Their synthesis is very similar to Kitamura’s; however, rather than using an anionic nucleophile, their nucleophile is neutral. Their strategy worked very well with pyridine, generating *N*-alkynyl pyridiniums **58a** and **58b** in 92% and 69% yields, respectively. When applying their strategy to azole nucleophiles, they were able to isolate *N*-alkynyl imidazolium **58c** in a 50% yield and *N*-alkynyl pyrazolium **58d** in a 72% yield, however they did have to increase the temperature for product **58d**. The researchers also mention that several pyridines with electron-withdrawing groups failed to give the desired products.

### 2.3. Cross-Coupling

Metal-catalyzed cross-coupling as a strategy for synthesizing *N*-alkynyl azoles has become increasingly popular in recent years due to the improved functional group tolerance over elimination methods and the general ease in which these reactions can be carried out. In many publications, the synthesis of *N*-alkynyl azoles are often included as part of the substrate scope and are not the primary focus of the paper. The following sections cover any paper in which the synthesis of an *N*-alkynyl azole is reported, either when it is the main focus of the paper or as part of a screen of the substrate scope.

#### 2.3.1. 1-Bromoalkynes

**Scheme 17:**
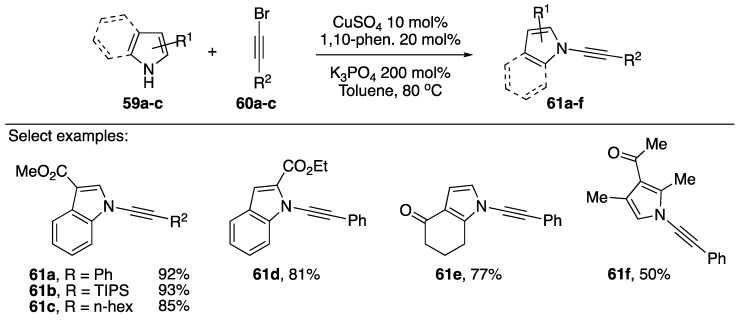
Hsung’s copper-catalyzed synthesis of various *N*-alkynyl azoles.

Many cross-coupling partners have been reported/evaluated for the synthesis of *N*-alkynyl azoles, but 1-bromoalkynes are used the most frequently. In 2003, Hsung reported the first metal-mediated synthesis of *N*-alkynyl amides, inspired by the work Buchwald and Hartwig. Hsung initially employed CuCN and 1-bromoalkynes [36]. The following year, in 2004, Hsung reported an improved procedure for the synthesis of *N*-alkynyl amides and included several *N*-alkynyl azoles, the first reported metal-catalyzed synthesis of *N*-alkynyl azoles (Scheme 17) [37]. The improved conditions involved CuSO_4_ as a precatalyst and 1,10-phenanthroline as a ligand and much lower temperatures. Hsung suggests these conditions are more environmentally friendly since they avoid using CuCN and copper halides.

**Scheme 18:**
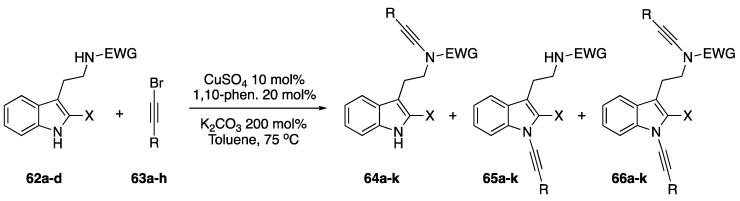
Competing alkynylation in tryptamine derivatives.

In 2006, as part of Hsung’s continued research into the chemistry of *N*-alkynyl amides, his group reported on their investigations into the competing alkynylation of a series of tryptamine derivatives (Scheme 18) [38]. The researchers were investigating whether *N*-alkynyl amides of tryptamine derivatives (**62a**–**d**) could be used in the synthesis of desbromoarborescidine A and C and were intrigued to find that these compounds also formed *N*-alkynyl indoles (**65a**–**k**) and bis-*N*-alkynylated products (**66a**–**k**) when subjected to their coupling conditions. Hsung suggests that similar pK_a_ values between the indole and amide could be leading to the mixtures in *N*-alkynylated products. When testing this theory by using stronger electron-withdrawing groups on the amine they observed that the competing alkynylation of the indole nitrogen completely disappears. Remarkably, the researchers also found that when using carbamate as the protecting group that the *N*-alkynyl indole product was exclusively formed.

**Scheme 19:**
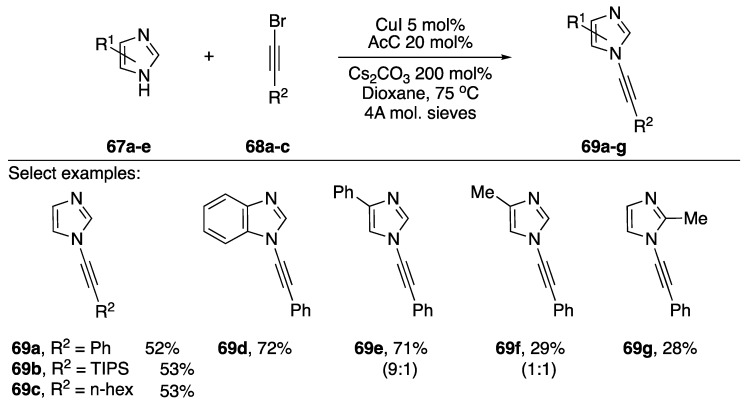
Kerwin’s copper-catalyzed synthesis of *N*-alkynyl imidazoles.

In 2008, Kerwin published a cross-coupling of imidazoles and bromoalkynes (Scheme 19) [39]. This report constitutes the first example of a dedicated procedure for the metal-catalyzed synthesis of an *N*-alkynyl azole. 

The researchers found that when trying to synthesize the desired *N*-alkynyl imidazoles using Hsung’s [37] procedure or analogous procedures for preparing *N*-alkynyl amides [40,41] they only isolated trace amounts of the desired compounds were obtained. However, following a screen of reaction conditions they found that they could synthesize *N*-alkynyl imidazoles (**69a**–**g**) in moderate to good yields (28–72%) by employing 2-acetylcyclohexanone (AcC) as ligand and CuI as copper source. A variety of bromoalkynes worked in this reaction, and the conditions worked for benzimidazoles as well. In the case of 4-substituted imidazoles, Kerwin found that the reaction gave mixtures of *N*-alkynylated products; 4-methyl-1*H*-imidazole giving a 1:1 mixture of *N*-alkynyl imidazoles **69f**.

**Scheme 20:**
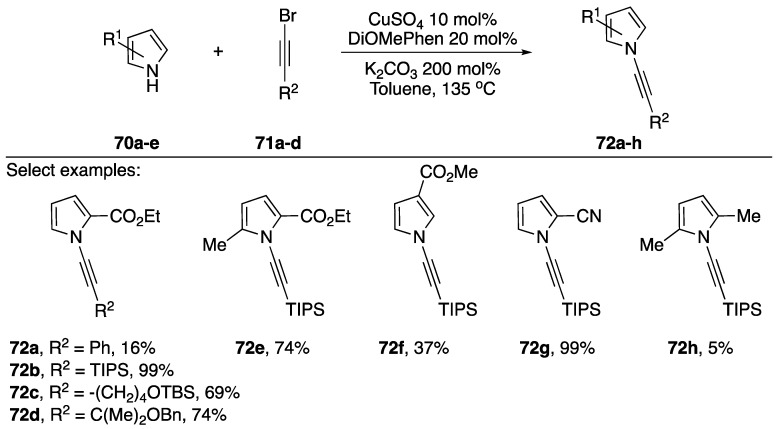
Kerwin’s copper-catalyzed synthesis of *N*-alkynyl pyrroles.

As part of their continued interest in the synthesis and reactivity of various *N*-alkynyl azoles, Kerwin has also published a synthesis of *N*-alkynyl pyrroles in 2017 (Scheme 20) [42]. The researchers were interested in synthesizing 2-substituted *N*-alkynyl pyrroles and found that the established methods for *N*-alkynylation were insufficient. They found that by increasing the temperature and using the more electron rich 4,7-dimethoxy-1,10-phenanthroline (DiOMePhen) ligand that they were able to synthesize various *N*-alkynyl pyrroles in poor to excellent yields. Kerwin found that the success of the pyrrole substrate **70** seemed to have a strong correlation with the nature of its substitution. Electron-rich (and sterically hindered) 2,5-dimethypyrrole gave only 5% of the desired *N*-alkynyl pyrrole **72h**, while pyrroles substituted with electron-withdrawing groups often gave near quantitative conversion (compounds **72b, 72g**).

**Scheme 21:**
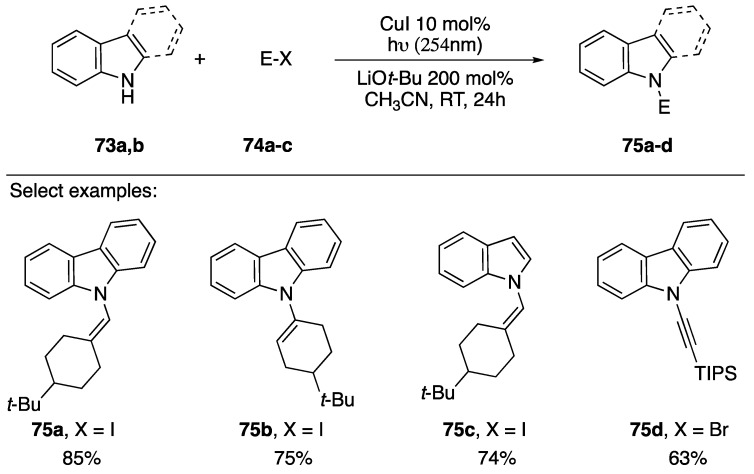
Peters and Fu’s photoinduced-copper-catalyzed synthesis of *N*-alkynyl carbazole.

In 2013, as part of their research into a room temperature photo-induced Ullmann coupling, Fu and Peters reported the synthesis of an *N*-alkynyl carbazole derivative in conjunction with several other substituted carbazoles (Scheme 21) [43]. The researchers discuss the benefits of copper-catalyzed C-N bond formation, but suggest one of the biggest downfalls is the often-required elevated reaction temperatures. Their work circumvents elevated reaction temperatures by using 254 nm light to excite the copper catalyst. Their method was applicable to the coupling of various vinyl iodides with carbazole and indole (**75a**–**c**), and even worked for the *N*-alkynylation of carbazole with a 1-bromoalkyne (**75d**).

**Scheme 22:**
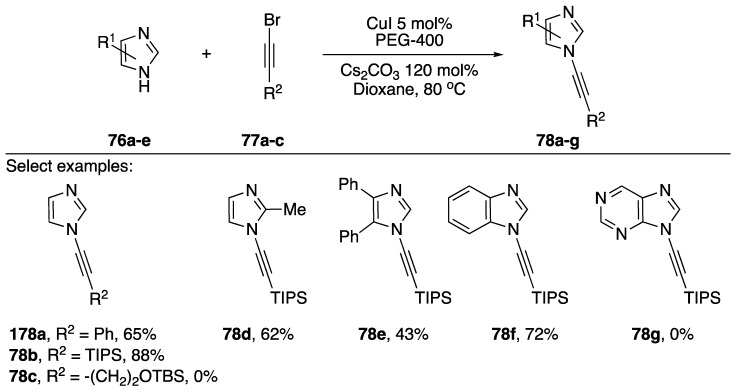
Burley’s microwave-promoted copper-catalyzed synthesis of *N*-alkynyl azoles.

Burley and researchers published a microwave-promoted copper-catalyzed synthesis of *N*-alkynyl imidazoles, benzimidazoles, pyrazoles, and indazoles in 2010 (Scheme 22) [44]. The researchers found that when using PEG-400 as a phase-transfer catalyst that the reaction times for the coupling were significantly reduced and the typical reaction was complete in only 30 min. Additionally, the researchers found that PEG-400 was also acting as a ligand for the copper catalyst and negated the need for additional ligands such as phenanthroline or acetylcyclohexanone (AcC). Using their method they synthesized various *N*-alkynyl imidazoles and benzimidazoles **74a**–**g** in moderate to good yields (43–88%). They did find that a 1-bromoalkyne containing a TBS ether failed to give the desired product **74c** and that their conditions failed to provide *N*-alkynyl purine **74g**. The researchers extended their method towards the synthesis of various *N*-alkynyl pyrazoles and indazoles.

**Scheme 23:**
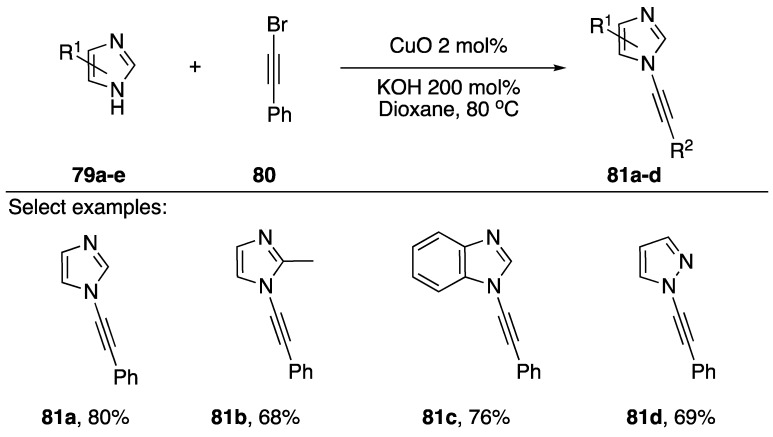
Das’s ligand-free copper-catalyzed synthesis of *N*-alkynyl azoles.

In 2011, Das also published a “ligand-free” synthesis of *N*-alkynyl azoles (Scheme 23) [45]. Unlike Burley’s protocol, which employed PEG-400 as a ligand for copper, this method has no exogenous ligand; however, the substrate azoles are well known to serve as ligands for copper. Also unique to their strategy, Das uses CuO as a source of copper and KOH as their base. The researchers report isolating *N*-alkynyl imidazoles, benzimidazoles, and pyrazoles **81a**–**d** in good yields (68–80%).

**Scheme 24:**
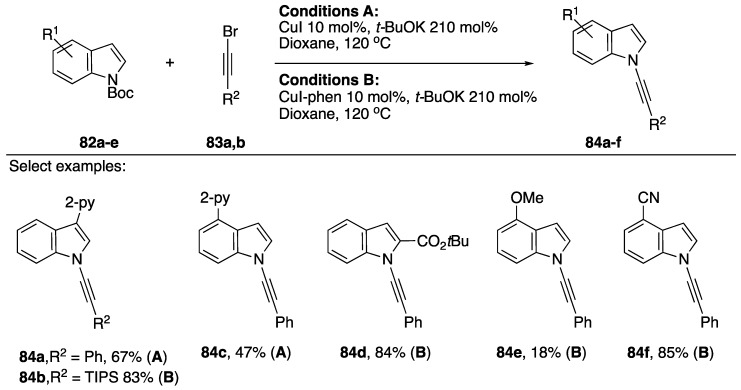
Wu’s copper-catalyzed *N*-alkynylation of Boc-protected indoles.

In 2013, Wu reported the *N*-alkynylation of indole from Boc-protected indoles (Scheme 24) [46]. The researchers were interested in synthesizing 2-alkynyl indoles directed by a 2-pyridyl group in the 3-position of the indole, and they were surprised when they recovered the deprotected and *N*-alkynylated indole product instead. Wu suggests this strategy could be synthetically useful since it is very common to employ Boc-protecting groups in the synthesis of indoles, a strategy circumventing the deprotection could improve the efficiency of the synthesis of *N*-alkynyl indoles derived from such compounds. They found that when there was a pyridine present on their starting indole (**78a**–**e**) that they didn’t have to use additional ligand for the synthesis to be successful (Conditions A). In other cases, they used 1,10-phenanthroline as a ligand (Conditions B). Wu’s strategy was successful at synthesizing a variety of *N*-alkynyl indoles both electron-rich and electron-deficient and with a variety of substitution patterns (**80a**–**f**), they also demonstrated the application of this strategy towards the synthesis on *N*-alkynyl pyrrole and *N*-alkynyl benzimidazole.

**Scheme 25:**
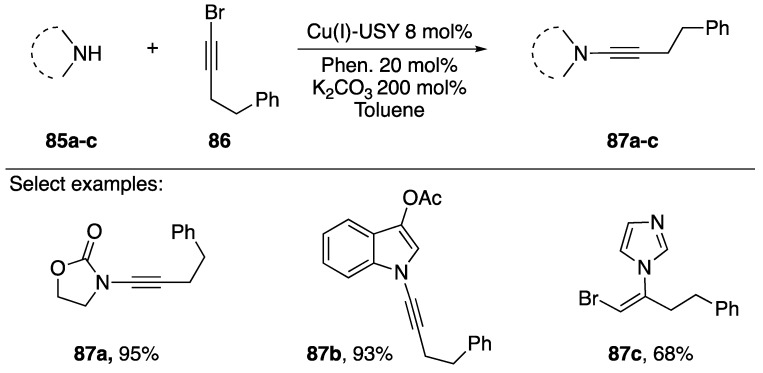
Pale’s copper-zeolite-catalyzed synthesis of *N*-alkynyl amides.

Pale and coworkers published a copper-zeolite based synthesis of *N*-alkynyl amides in 2014 in which they discuss its application to the synthesis of an *N*-alkynyl indole (Scheme 25) [47]. The researchers use a Cu(I)-ultra stable Y Zeolite (Cu(I)-USY) as their catalyst. Interestingly, by using a zeolite catalyst Pale was able to recover and recycle the catalyst, showing that after reusing the catalyst five times they only observed a marginal decline in activity (~15%). The majority of substrates screened by the researchers were amides such as **87a**, however they demonstrated the use of their methodology towards the synthesis of *N*-alkynyl indole **87b** and showed in the case of imidazole that they isolated the vinyl-bromide **87c** as the only product.

**Scheme 26:**
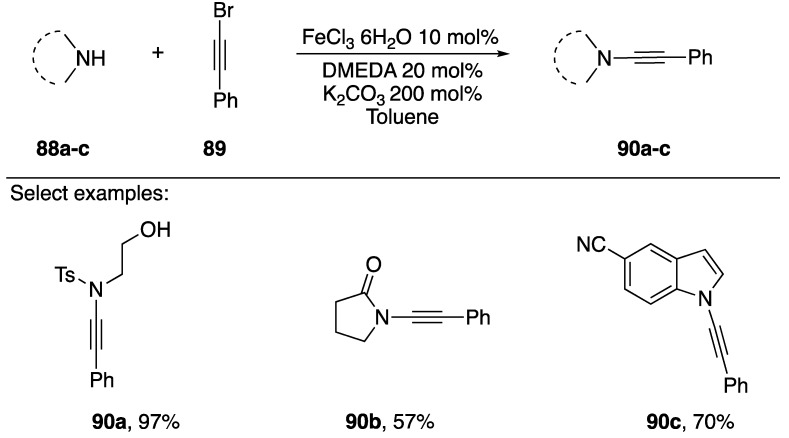
Zhang’s iron-catalyzed synthesis of *N*-alkynyl amides.

In 2009, Zhang reported an iron-catalyzed synthesis of *N*-alkynyl amides using 1-bromoalkynes as a coupling partner (Scheme 26) [48]. This is the first synthesis of *N*-alkynyl amides using an iron catalyst and the only instance in which a non-copper catalyst has been used in the synthesis of *N*-alkynyl azoles. In general, the researchers reported yields that were comparable to other published methods, synthesizing a tosyl-amide substrate **86a** in a 97% yield and an *N*-alkynyl lactam **86b** in a 57% yield. They only applied their conditions to the synthesis of one *N*-alkynyl azole, **86c**. Interestingly, Zhang was able to recover and reuse the iron catalyst, showing that after recycling for nine runs the yields only dropped by 15%.

#### 2.3.2. 1,1-Dibromoalkenes

**Scheme 27:**
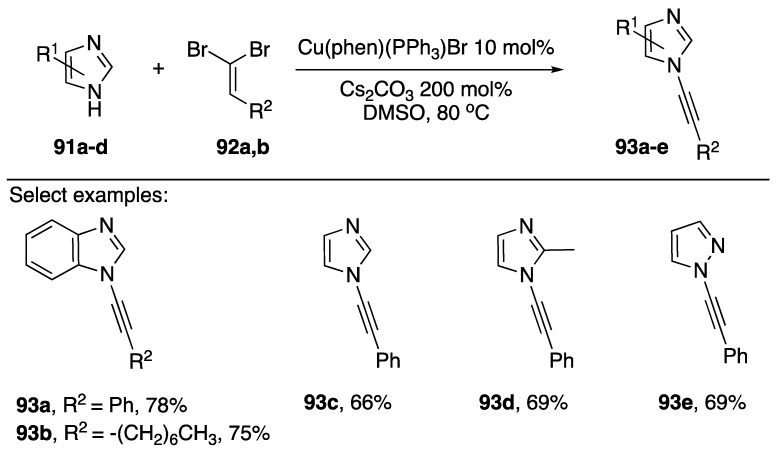
Das’s [Cu(Phen)PPh_3_Br]–catalyzed synthesis of *N*-alkynyl azoles.

In addition to 1-bromoalkynes as cross-coupling partners for the synthesis of *N*-alkynyl azoles, 1,1-dibromoalkenes have found use as competent coupling partners. For certain substrates, using a 1,1-dibromoalkene as a coupling partner would be advantageous due to their ease of synthesis. For example, rather than converting an aldehyde into an alkyne then subsequently the 1-bromoalkyne, researchers can simply make the analogous 1,1-dibromoalkene in one step from the same aldehyde. In 2011, Das published a paper reporting the coupling of various 1,1-dibromoalkenes with azoles (Scheme 27) [49]. This report was closely followed by two similar reports by Shang [50] and Evano [51]. Das reports that Cu(phen)(PPh_3_)Br in DMSO with Cs_2_CO_3_ as base provides *N*-alkynyl azoles **89a**–**e** in good yields (66–78%). Interestingly this report is the only example of a phosphine ligand being used in the metal-catalyzed synthesis of an *N*-alkynyl azole. In their paper, the researchers suggest two possible mechanistic pathways leading to the observed alkynylated products: (1) coupling occurs with the dibromide to give an *N*-alkenyl azole followed by elimination to the alkyne, or (2) the 1,1-dibromoalkene first undergoes conversion to the corresponding 1-bromoalkyne and then cross-coupling occurs between the 1-bromoalkyne and the azole. Das favors the second mechanism.

#### 2.3.3. Alternative Coupling Partners

**Scheme 28:**
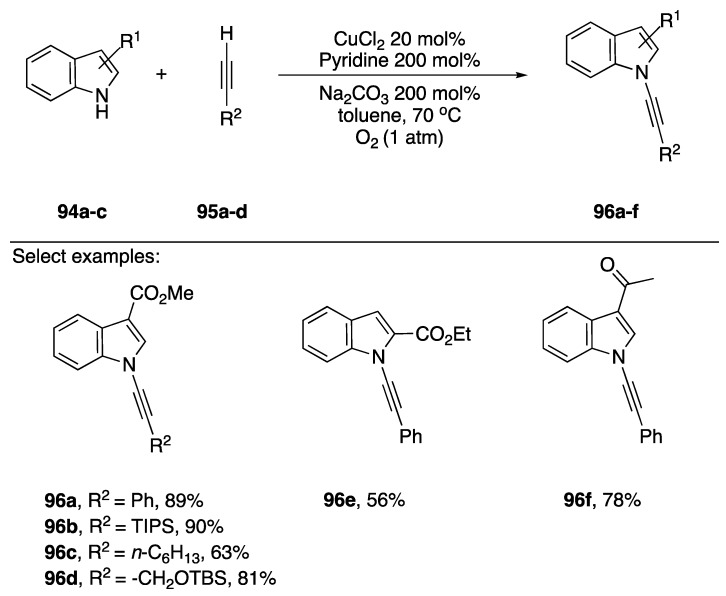
Stahl’s oxidative copper-catalyzed synthesis of *N*-alkynyl indoles.

Terminal alkynes have also been used as cross-coupling partners in the synthesis of *N*-alkynyl azoles. The advantage of using a terminal alkyne is avoiding the additional step to introduce the 1-bromo group and avoiding the use of halogens all together, a more atom efficient process. In 2008, Stahl reported using an oxidative *N*-alkynylation strategy for the synthesis of *N*-alkynyl amides and several *N*-alkynyl indoles (Scheme 28) [52]. Stahl, inspired by the Glaser-Hay coupling [53] and the Chan-Lam coupling [54] found that under the right conditions they could limit the Glaser-Hay homocoupling of the alkynes and limit the formation of 1-chloroalkynes, isolating the desired *N*-alkynylated products in high yields. Several indoles **94a**–**c** were tolerated under these conditions and aromatic, alkyl, and silyl alkynes **95a**–**d** were all tolerated as well. In 2015, Troung published a similar strategy for the synthesis of *N*-alkynyl oxazolidinones, however they used a copper metal-organic-framework (MOF) [55]. While their method performed well in preparing *N*-alkynyl oxazolidinones (31–96% yield), application to an indole substrate afforded **96f** in only 32% yield.

**Scheme 29:**
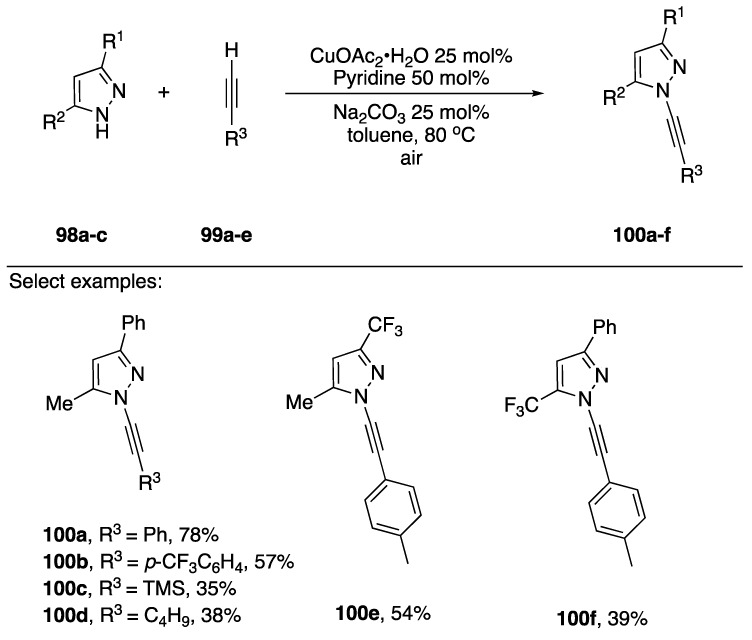
Bhattacharjee’s oxidative copper-catalyzed synthesis of *N*-alkynyl pyrazoles.

In 2017, Bhattacharjee and co-workers reported the oxidative coupling of terminal acetylenes and pyrazoles (Scheme 29) [56]. They employed similar conditions as Stahl, but they used copper (II) acetate hydrate as the copper source and air as oxidant. 

A series of 3,5-disubstiuted pyrazoles **94a**–**c** were coupled to terminal acetylenes in good to modest yield to afford the regioisomerically pure *N*-alkynyl pyrazoles **100a**–**f**. The authors note that yields drop when alkyl substituted acetylenes are employed.

**Scheme 30:**
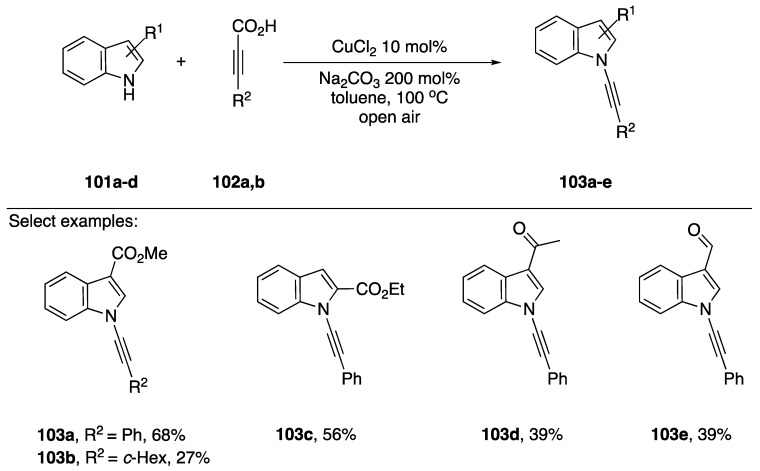
Jiao’s copper-catalyzed decarboxylative synthesis of *N*-alkynyl indoles.

In 2010, Jiao published a decarboxylative coupling strategy for preparing *N*-alkynyl amides and azoles (Scheme 30) [57]. This strategy benefits from not using halide containing starting materials, and has the added benefit that the formation of Glaser-Hay oxidative dimerization products is minimized. Additionally, many alkynyl carboxylates are readily available. The researchers showed that this strategy could be used to effectively synthesize many *N*-alkynyl indole substrates **103a**–**e**, even tolerating an aldehyde. Unfortunately, when compared to other methodologies, *vide supra*, Jiao’s decarboxylative-coupling strategy often falls short in terms of yield, with yields ranging from 27% to 68%.

### 2.4. Other Methods

**Scheme 31:**
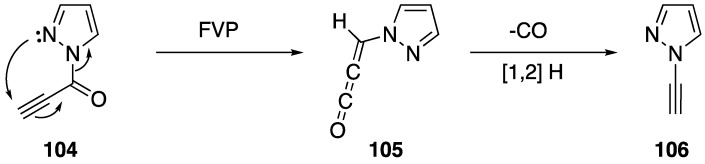
Brown’s flash vacuum pyrolysis synthesis of *N*-alkynyl pyrazole.

One of the more unusual syntheses of an *N*-alkynyl azole is Brown’s synthesis of *N*-ethynylpyrazole (**106**) (Scheme 31) [58]. During the course of their research studying the flash vacuum pyrolysis of 1-alkynoyl-3-methylpyrazoles into pyrazolo[1,5-*a*]pyridine-5-ols, the researchers found that substrates lacking a methyl substituent in the 3-position formed *N*-alkynyl pyrazole in low yields. Brown proposes a mechanism that proceeds through an allene-ketene intermediate, such as **105**. He suggests when a substrate lacks methyl group in the 3-position that the intermediate loses carbon monoxide and forms a vinylidene carbene that rearranges to the *N*-alkynyl pyrazole.

## 3. Reactions of *N*-Alkynyl Azoles

*N*-alkynyl azoles, in a similar manner to *N*-alkynyl amides, are characterized by their polarized alkyne due to the attached nitrogen, which is moderated by the participation of this nitrogen in the aromatic azole ring. This alkyne polarization, with the β-carbon becoming more nucleophilic and the α-carbon becoming more electrophilic, leads to distinct reactivity. As interest in the synthesis of *N*-alkynyl azoles has grown over the past decade, interest in the reactions of *N*-alkynyl azoles has grown as well. The following sections will discuss the many different reactions of *N*-alkynyl azoles that have been reported. In many cases, the reactions of *N*-alkynyl azoles are analogous to those of *N*-alkynyl amides. Publications reporting reactions of *N*-alkynyl amides often times include one or two examples of *N*-alkynyl azoles, typically *N*-alkynyl indoles, in sections describing the scope of the reaction. These are included in this section, with notes indicating this. In addition, there are a number of reactions specific to *N*-alkynyl azoles, or reactions that have only been studied in *N*-alkynyl azoles, which are also highlighted in this section.

### 3.1. Reactions of Alkyne C-H

**Scheme 32:**
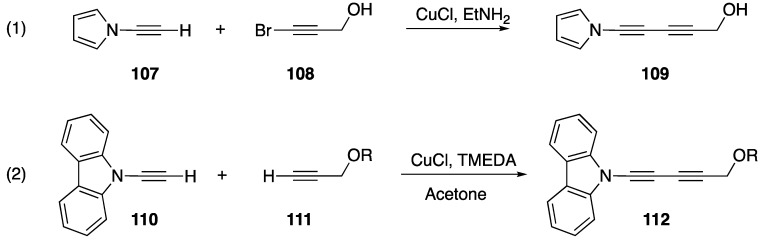
Synthesis of diacetylenes of *N*-alkynyl azoles.

Terminal alkynes, including *N*-alkynyl amides, participate in nucleophilic addition and coupling reactions [59], and similar reactions have also been reported for *N*-alkynyl azoles. In 1992, Paley published one of the first reactions of an *N*-alkynyl azole (Scheme 32, Reaction (1) [14]). In their publication they report the synthesis of diacetylene **109** from *N*-alkynyl pyrrole **107** and 1-bromoalkyne **108** using a Cadiot-Chodkiewicz coupling. This report provides a first glimpse into the general reactivity and stability of *N*-alkynyl azoles and products derived from *N*-alkynyl azoles. The researchers reveal that diacetylene **109** undergoes spontaneous polymerization upon formation. In 2016, Okuno reported a very similar reaction with *N*-alkynyl carbazole **110** (Scheme 32, Reaction (2) [60]). In their work, the analogous diacetylene **112** was more stable and Okuno was able to isolate the compound.

**Scheme 33:**
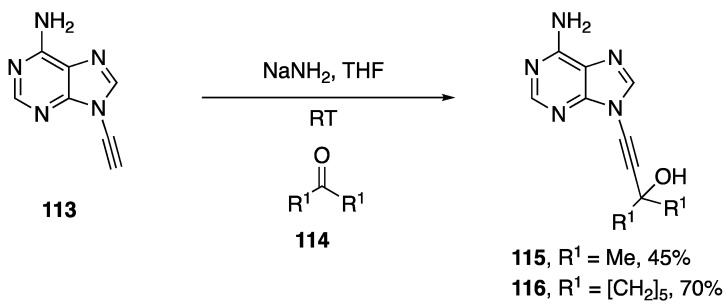
Zemlicka’s synthesis of carbinols of *N*^9^-alkynyl adenine.

In 1994, as part of his publication disclosing the synthesis of various *N*-alkynylated purines and pyrimidines, Zemlicka investigated the addition of *N*^9^-alkynyl adenine to various ketones (Scheme 33) [16]. 

The researchers discuss the importance of base on the outcome of the reaction, in the case of LiNH_2_ and *n*-BuLi the desired carbinols were not isolated. However, when NaNH_2_ was used as base, the carbinols of acetone (**115**) and of cyclohexanone (**116**) were isolated in 45% and 70% yield, respectively.

**Scheme 34:**
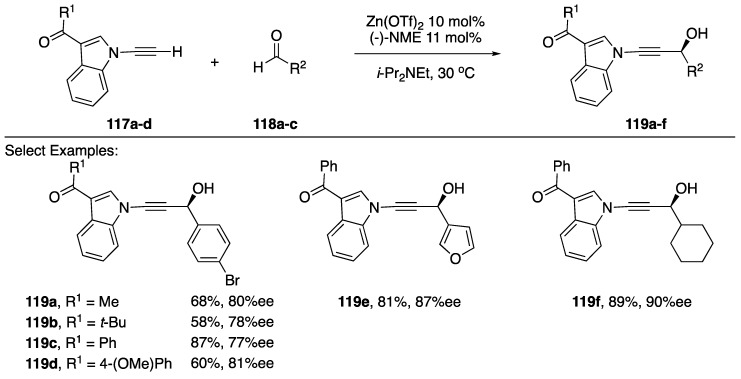
Wolf’s enantioselective addition of *N*-alkynyl indole to various aldehydes.

In 2014, Wolf reported a zinc-catalyzed enantioselective addition of *N*-alkynyl amides and indoles to aldehydes (Scheme 34) [61]. This publication represents the first example of a method for adding an *N*-alkynyl azole or *N*-alkynyl amide to a carbonyl electrophile without relying on a strong base to deprotonate the alkyne. Additionally, using (-)-NME as a ligand to the Zn(OTf)_2_ catalyst, the researchers were able to generate high levels of enantiomeric excess in the products (77–90% ee). While the scope of the indole substrate wasn’t probed extensively by Wolf, limited to 3-substituted indoles, a variety of aldehydes were tolerated in this reaction; including: benzaldehydes, furan-3-carbaldehyde, and sterically hindered aliphatic aldehydes, which afford the corresponding propargylic alcohols **119a**–**f** in moderate to good yields (58–89%).

**Scheme 35:**
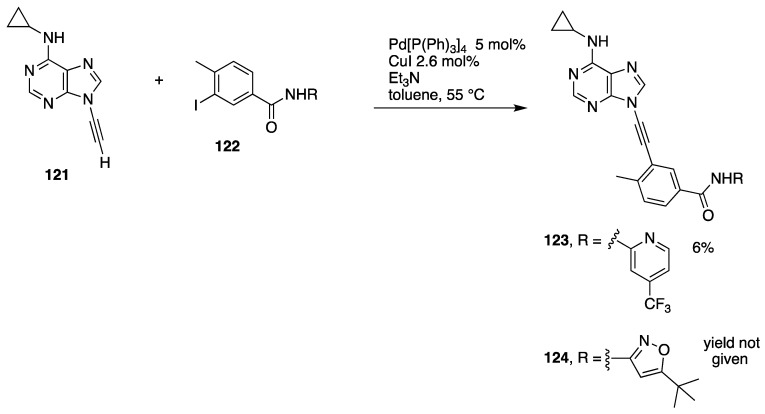
Huang’s low-yielding Sonogashira coupling of an *N*-alkynyl purine.

The participation of terminal *N*-alkynyl azoles in Sonogashira coupling reactions can be complicated by alternative, palladium-catalyzed hydroalkynylation reactions (see Section 3.2.2). However, as part of their work investigating the structure-activity relationship of ethynyl purines and analogues as BCL-ABL inhibitors, Huang and co-workers at ARIAD Pharmaceuticals prepared a series of *N*^9^-alkynyl purines **123** and **124** by Sonogoshira coupling of the *N*-ethynyl purine **121** with the aryl iodides **122**, albeit in very low yield (Scheme 35) [62].

### 3.2. Addition Reactions

**Figure 2:**
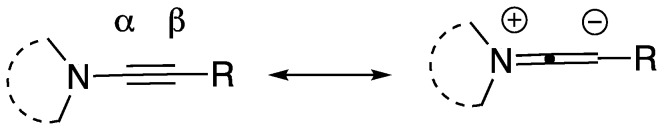
Polarization of the *N*-alkynyl azole triple bond.

The polarization of the alkyne of *N*-alkynyl amides and *N*-alkynyl azoles (Figure 2) gives rise to regioselective addition reactions, and these reactions are one of the most explored classes of reactions of these compounds. In this section, we describe the wide variety of addition reactions, both intermolecular and intramolecular, that have been reported in the literature for *N*-alkynyl azoles. Cycloaddition, annulation, and other reactions involving the triple bond of *N*-alkynyl azoles are treated in separate sections.

#### 3.2.1. Carbon-Heteroatom Bond Formation

**Scheme 36:**
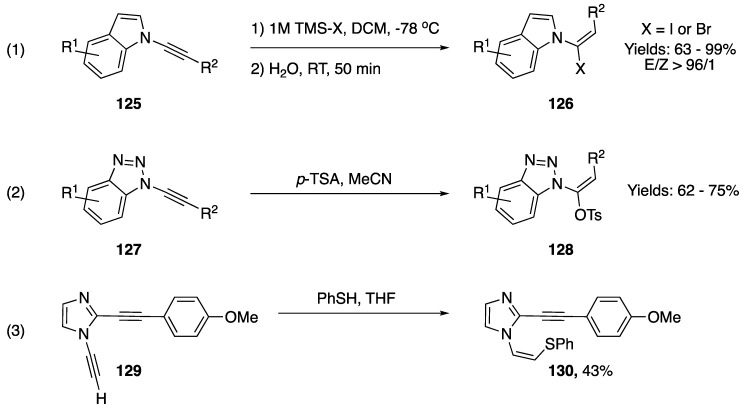
Addition of acids and thiophenol to various *N*-alkynyl azoles.

Addition reactions resulting in the formation of a new carbon-heteroatom bond are very prevalent among *N*-alkynyl azoles. The addition of Bronsted acids, a classic reaction of alkynes, has been reported on several occasions (Scheme 36). In 2013, Iwasawa reported a method for the stereoselective synthesis of 1-(1-halovinyl)-1*H*-indoles from their respective *N*-alkynyl indoles via the addition of in situ generated HX acids (Scheme 36, equation. 1) [63]. The researchers used TMS-X and water to generate HX in a controlled manner, and were able achieve excellent levels of stereoselectivity and isolated the vinyl-halides in good yields. In 2000, Katritzky reported the addition of *p*-toluenesulfonic acid to *N*-alkynyl benzotriazoles as part of a methodology for the homologation of carboxylic acids (Scheme 36, equation 2) [17]. The researchers isolated vinyl-tosylates, analogous compounds to those synthesized by Iwasawa, in moderate yields. In 2011, as part of a study on an aza-Bergman rearrangement of 1,2-dialkynylimidazole Kerwin reported an addition of thiophenol to an *N*-alkynyl imidazole (Scheme 36, equation 3) [64]. Interestingly, in this example the sulfur nucleophile adds to the β-carbon of the alkyne to give **130** in a 43% yield.

**Scheme 37:**
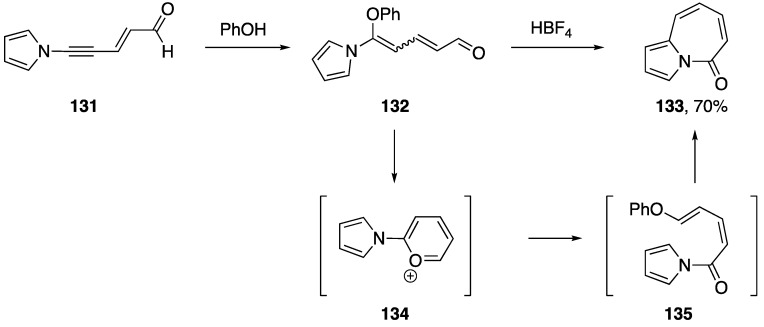
Neuenschwander’s rearrangement of 5-substituted 5-aminopentadienals.

In 1998, while studying the rearrangement of 5-substituted 5-aminopentadienals, Neuenschwander reported an interesting transformation of an *N*-alkynyl pyrrole following the addition of phenol to the alkyne (Scheme 37) [65]. The researchers found that treating *N*-alkynyl pyrrole **131** with phenol gave the addition product **132** and that upon further treatment with HBF_4_, they isolated the pyrrole **133** in 70% yield.

**Scheme 38:**
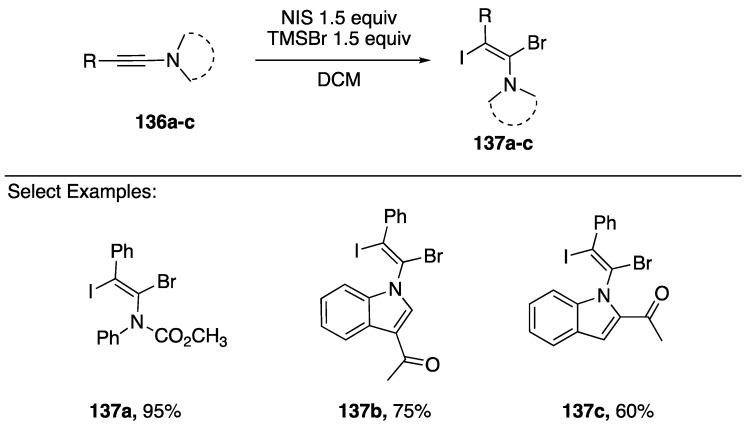
Regio- and stereoselective iodobromination of *N*-alkynylindoles.

Additions of halogens and pseudohalogens to *N*-alkynyl azoles can occur with high regio- and stereoselectivity due to the polarized nature of the alkyne. In 2014, Iwasawa reported iodobromination of a series on *N*-alkynyl amides and *N*-alkynylindoles **136a**–**c** (Scheme 38) [66]. These reactions afford exclusively the (*E*)-1-bromo-2-iodoalkenes **137a**–**c** in good yields from in situ generated IBr using NIS and a silylbromide.

**Scheme 39:**
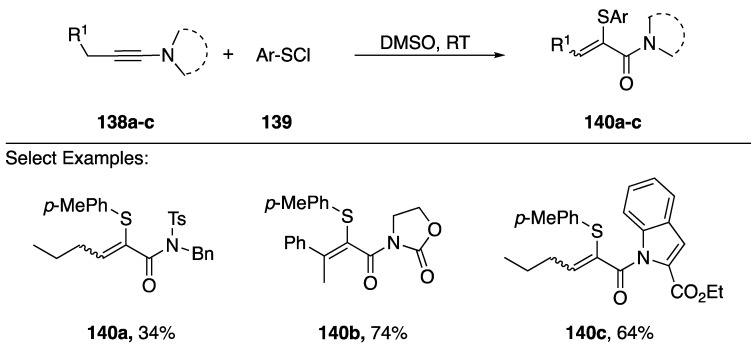
Zhu’s sulfenylchloride-mediated addition of DMSO to *N*-alkynyl amides.

In 2016, Zhu reported a sulfenylchloride-mediated addition of DMSO to *N*-alkynyl amides and azoles generating acrylamides with sulfur substitutions in the α-position (Scheme 39) [67]. This method worked for various *N*-alkynyl amides **140a,b** and also worked with *N*-alkynyl indole **149c**. The researchers suggest that initially a sulfonium ion forms between the alkyne and sulfenylchloride, which undergoes subsequent addition of DMSO to generate the observed products. In addition to sulfur, Zhu demonstrated an analogous synthesis of α-selanyl and α-tellanyl acrylamides.

**Scheme 40:**
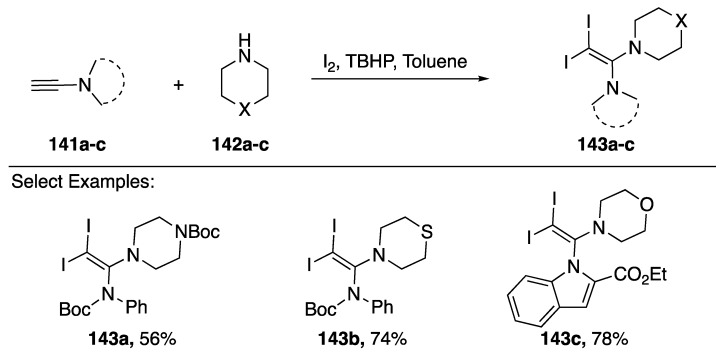
Zhu’s iodoamination of *N*-alkynyl amides and indole.

Iodine-promoted additions of heteronucleophiles to alkenes and alkynes represent a classic method for constructing carbon-heteroatom bonds [68]. An interesting iodoamination of *N*-alkynyl amides and *N*-alkynyl azoles was reported by Zhu in 2016 (Scheme 40) [69]. The researchers found that when treating an *N*-alkynyl amide/azole **141a**–**c** with a cyclic secondary amine **142a**–**c** in the presence of iodine and tert-butyl hydrogen peroxide they could synthesize vinyl-diiodides **143a**–**c** in good yields. Zhu suggests that under these conditions a 1-iodoalkyne initially forms, followed by an iodine-mediated addition of the nucleophile to give the observed products.

**Scheme 41:**
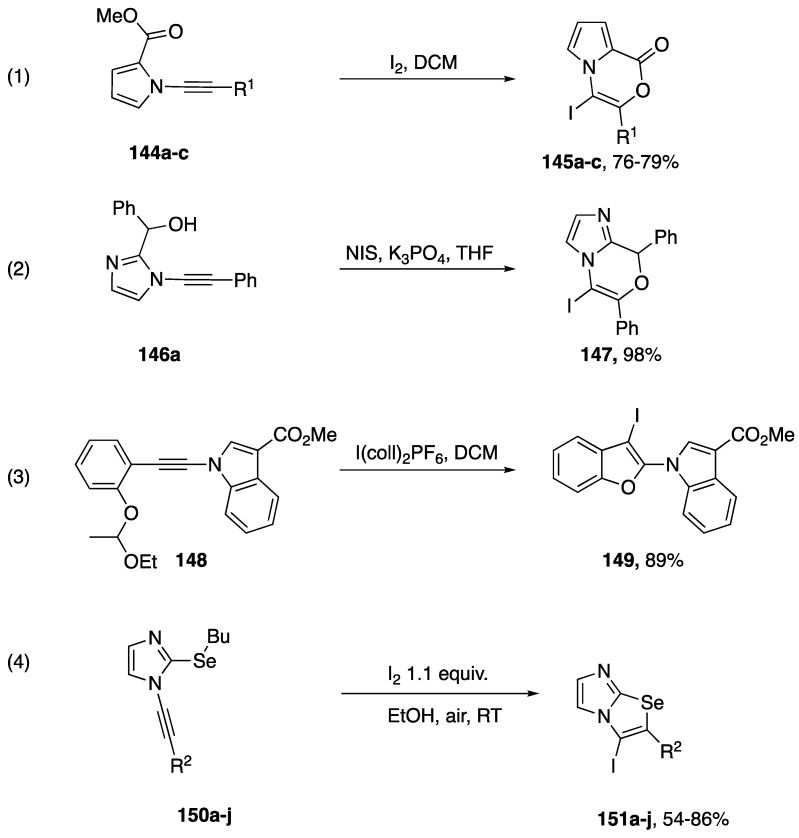
Iodocyclizations of *N*-alkynyl azoles.

**Scheme 42:**
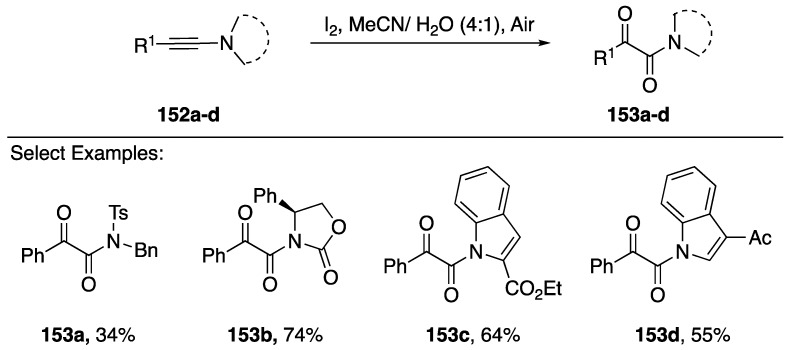
Zhu’s iodine-mediated oxidation of *N*-alkynyl amides and indoles.

There have been several reports of iodocyclizations of *N*-alkynyl azoles published in the literature (Scheme 41). In 2017, as part of their paper investigating the intramolecular addition of hydrazide to *N*-alkynyl pyrrole (*vide infra*), Balci reported an iodocyclization from *N*-alkynyl pyrrole starting materials **144a**–**c** (Scheme 41, Reaction (1) [70]). The researchers noted that all of the substrates they investigated underwent a 6-*endo*-*dig* cyclization to give the corresponding vinyl-iodides. DFT calculations performed by Balci predicted that the iodonium intermediate should carry more positive charge on the β-carbon of the alkyne, providing an explanation for the observed regioselectivity. Kerwin disclosed an iodocyclization in 2009 (Scheme 41, Reaction (2) [71]). They reported a single example of the *N*-iodosuccinimide mediated intramolecular addition of an alcohol to an *N*-alkynyl imidazole; isolating vinyl iodide **147a** in a 98% yield. Interestingly, iodide **147a** is also the result of a 6-*endo-dig* cyclization. In 2014, Okitsu published a more general iodocyclization of various *N*-alkynyl amides, including an example of an *N*-alkynyl indole (Scheme 41, Reaction (3) [72]). Okitsu uses I(coll)_2_PF_6_ as their source of iodine and ethoxyethyl ethers as the nucleophile. Amazingly, the reactions required less than 5 s to run to completion and give the substituted benzofuran product **149** in excellent yield. In 2013, Zeni published an electrophilic cyclization of a series of *N*-alkynyl-2-(butylselenyl)imidazoles **150a**–**j** to imidazo[2,1-*b*][1,3]selenazoles **151a**–**j** (Scheme 42, equation 4) [73]. The corresponding 2-thiomethyl imidazoles also undergo cyclization to imidazo[2,1-*b*][1,3]thiazoles in good yield; however, cyclization of a *N*-alkynyl-2-(butylselenyl)benzimidazole was lower yielding.

Zhu has also reported an iodine-mediated oxidation of *N*-alkynyl amides/azoles (Scheme 42) [74]. Their method consists of treating the starting *N*-alkynes **152a**–**d** with I_2_ in an acetonitrile and water mixture, open to air. Under these conditions, the researchers isolated α-ketoamides **153a**–**d** in moderate to good yields (34–74%). Additionally, the reaction worked with both 2- and 3-substituted *N*-alkynyl indole starting materials. The researchers propose a reaction mechanism that proceeds initially through an iodine-promoted addition of water to the α-carbon of the alkyne, generating an α-iodo amide, and subsequently an oxidation to a ketone by air. Iwasawa also published in 2014 an NIS-mediated oxidation of *N*-alkynyl amides and an *N*-alkynyl indole, but their reported yield for **153c** is lower (34%) [75].

**Scheme 43:**
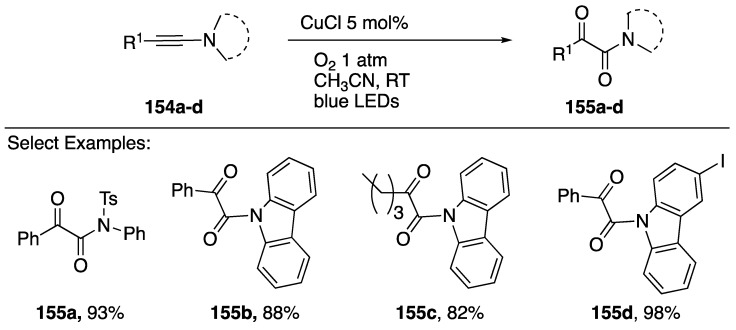
Hwang’s photochemical oxidation of *N*-alkynyl carbazoles.

Interestingly, an alternative route to these α-ketoamide products from *N*-alkynyl azoles was reported by Hwang in 2017 [76]. This method employs copper catalyst and irradiation with blue LEDs in the presence of 1 atm of O_2_ (Scheme 43). A series of *N*-alkynyl sulfonamides **155a** and carbazoles **155b**–**d** can be oxidized in good yields through a proposed photoexcitation of a Cu(I)-π complex (λ_max_ = 460 nm) leading to formation of a Cu(II) peroxo-complex intermediate.

**Scheme 44:**
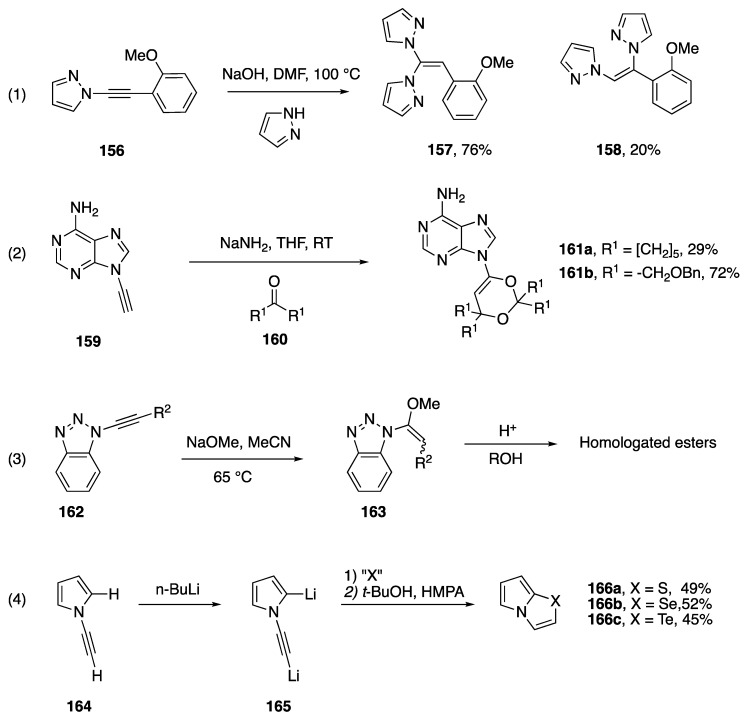
Additions to *N*-alkynyl azoles under basic conditions.

The addition of heteronucleophiles to *N*-alkynyl azoles has also been reported to occur under basic conditions. Zhang, Cui and co-workers investigated a copper-free coupling of 1,1-dihaloalkenes with excess azoles in the presence of NaOH to afford dihetaryl alkenes [77]. They propose a mechanism involving the formation on *N*-alkynyl azoles as intermediates (see Section 2.3.2), which undergo addition under the basic reaction conditions. They provided support for this mechanism by showing that when treated under these reaction conditions in the presence of excess pyrazole, the *N*-alkynyl pyrrole **156** affords the same products **157** and **158**, in similar ratio compared with the reaction of the corresponding 1,1,-dibromoalkene (Scheme 44, Reaction (1)). In 1994, Zemlicka reported the addition of oxygen nucleophiles to *N*^9^-alkynyl adenine (Scheme 44, Reaction (2) [16]). The heterocyclic products, **161a,b** were isolated when the researchers were trying to perform the 1,2 addition of the alkyne of **159** into various ketones. The author suggests that these compounds form when the initially formed propargylic alcohol adds to another equivalent of ketone forming a hemiketal, and the hemiketal oxygen proceeds to add to the alkyne. In 2000, Katritzky reported the addition of sodium methoxide to *N*-alkynyl benzotriazoles (**162**) within the paper describing their method for the homologation of carboxylic acids, treatment of the resultant addition product with acid gave homologated esters (Scheme 44, Reaction (3) [17]). While the reaction afforded only one regioisomer, ~80:20 mixtures of (*E*)- and (*Z*)-**163** were obtained. In 1995, Trofimov reported the reaction of dilithiated *N*-ethynyl pyrrole, **165**, with elemental sulfur, selenium, and tellurium (Scheme 44, equation 4) [78]. The researchers isolate the pyrrolo[2,1-b][1,3]chalcogenazoles **166a**–**c**, in modest yields. A similar cyclization of *N*-alkynyl imidazoles was reported in 2012 by Zeni [79].

**Scheme 45:**
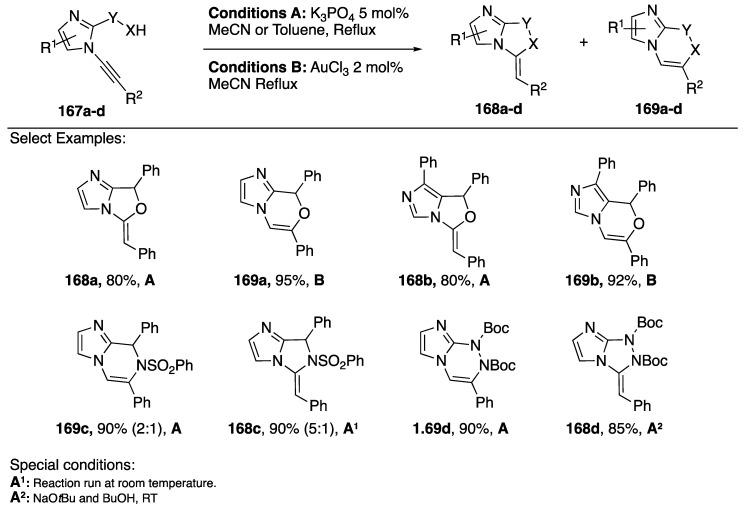
Kerwin’s regiocontrolled cyclization to imidazoazines and imidazoazoles.

In 2009, Kerwin reported a method for the synthesis of imidazo[1,2-c]-oxazoles and imidazo[2,1-c][1,4]oxazines starting from *N*-alkynyl imidazoles (Scheme 45) [71]. Using base catalyzed conditions, the researchers reported a 5-*exo*-*dig* cyclization and when AuCl_3_ was used as a catalyst the reaction proceeded via a 6-*endo*-*dig* cyclization (**168a** and **169a**). In a follow up report, Kerwin and researchers investigated the intramolecular addition of other nucleophiles; including: 5-hydroxymethyl-imidazoles, p-toluenesulfonamides, and Boc-protected hydrazine (Scheme 45) [80]. 

In this paper, they found that the new alcohol substrates reacted analogously to the previous alcohol additions (**168b** and **169b**); however, the nitrogen nucleophiles reacted less predictably. For both substrates containing nitrogen nucleophiles the researchers observed 6-*endo*-*dig* cyclizations when using conditions A, the same conditions that resulted in 5-*exo*-*dig* cyclizations with the oxygen nucleophiles **169c** and **169d**. Interestingly, gold catalysis yielded neither of the desired products. Ultimately Kerwin found that they could get the 5-*exo*-*dig* cyclization products with the nitrogen nucleophiles using base catalysis at room temperature (compounds **168c** and **168d**). The researchers note that the observed reaction temperature-dependent regioselectivity did not seem to be a kinetic versus thermodynamic effect.

**Scheme 46:**
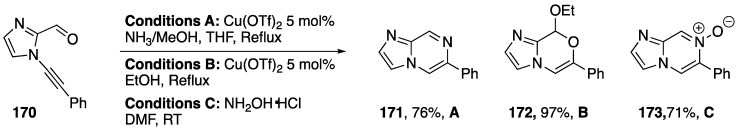
Kerwin’s synthesis of several imidazo-fused heterocycles.

In 2014, as part of the same paper, Kerwin reported the synthesis of several imidazo-fused heterocycles from 2-carbaldehyde-1-alkynylimidazole **170** (Scheme 46) [80]. Treatment of **170** with ammonia and catalytic Cu(OTf)_2_ gave imidazolopyrazine **171** in a 76% yield, while treatment with EtOH and Cu(OTf)_2_ gave the cyclic acetal derivative **172** nearly quantitatively. Remarkably, without the need for metal-catalysis, *N*-alkynyl imidazole **170** reacts with hydroxylamine to afford the cyclized product **173**, an imidazolopyrazine *N*-oxide, in 71% yield.

**Scheme 47:**
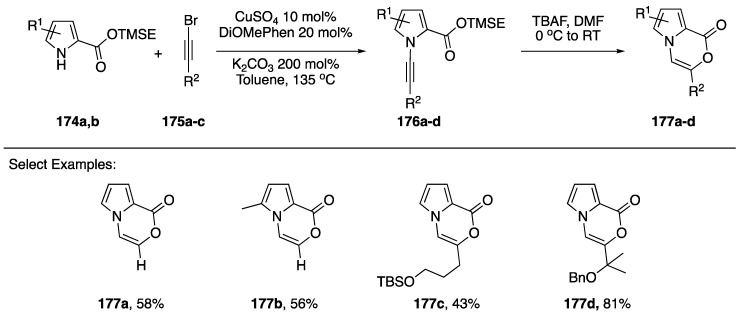
Kerwin’s synthesis of pyrrole[2,1-c]oxazin-1-ones.

In 2017, Kerwin reported the synthesis of pyrrole[2,1-c]oxazin-1-ones via the intramolecular addition of carboxylate nucleophiles to the alkyne of *N*-alkynyl pyrroles (Scheme 47) [42]. The researchers used trimethylsilylethyl (TMSE) esters as protected carboxylic acids to carry out the copper-catalyzed synthesis of *N*-alkynylazoles **176a**–**d**. Upon deprotection of the acids with tetra-n-butylammonium fluoride (TBAF) in DMF, the substrates underwent spontaneous 6-*endo*-*dig* cyclizations to the corresponding pyrrole[2,1-c]oxazin-1-ones **177a**–**d**. Several different substitutions on the alkyne and pyrrole were tolerated and the researchers even used this methodology to carry out the formal synthesis of peramine, a rye grass endophytic fungal alkaloid.

**Scheme 48:**
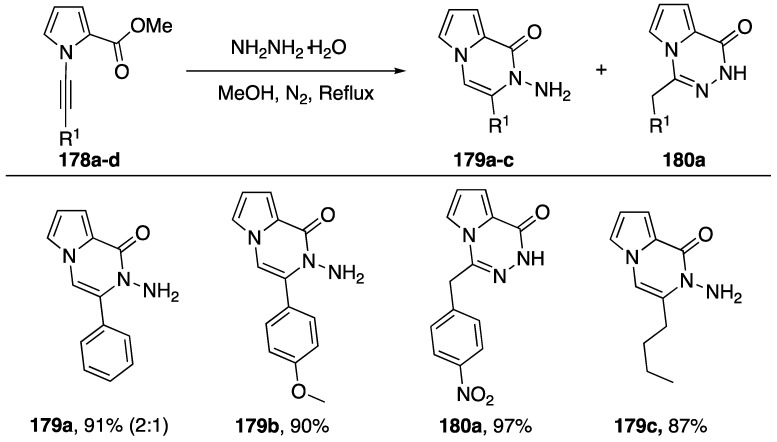
Balci’s intramolecular addition of hydrazide to *N*-alkynyl pyrrole.

Balci published an intramolecular addition of hydrazide to *N*-alkynyl pyrroles in 2017 (Scheme 48) [70]. Starting with *N*-alkynyl pyrroles **178a**–**d** substituted with a methyl ester in the 2-position the researchers refluxed the compounds with hydrazine giving the hydroamination addition products **179a**–**c** and **180a**. Balci suggests that a hydrazide is initially formed and subsequently adds to the alkyne. The researchers observed varying regioselectivies depending on the nature of the substitution on the alkyne. If the substitution was electron withdrawing, such as nitrobenzene, they recovered the α-addition product **180a**, but if the substitution was electron donating or aliphatic they observed β-addition (compounds **179b,c**) In the case of phenyl substitution, they isolated a mixture of the two regioisomers, favoring addition to the β-carbon.

**Scheme 49:**
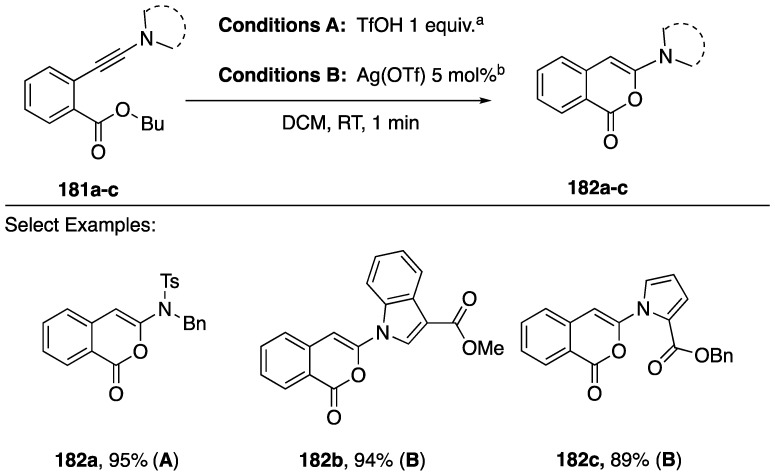
Gillaizeau’s cyclization of *ortho*-ynamidyl benzoate esters to 3-aminoisocoumarins.

In contrast to the predominant heteroatom additions to the β-position of *N*-alkynyl azoles in cyclizations under coinage metal catalysis (Scheme 45, Scheme 46 and Scheme 47), an alternative cyclization of *N*-alkynyl amides and azoles was reported by Gillaizeau in 2018 (Scheme 49) [81]. These workers describe the facile cyclization of *ortho*-ynamidyl benzoate esters **180a**–**c** to 3-aminoisocoumarins **181a**–**c** in the presence of an equivalent of a Bronsted acid or catalytic Lewis acid. The authors note that none of the 5-*exo-dig* cyclization products corresponding to addition to the β-carbon of the alkyne were observed, and that a range of *ortho*-ynamidyl het(aryl) esters also undergo cyclization.

**Scheme 50:**
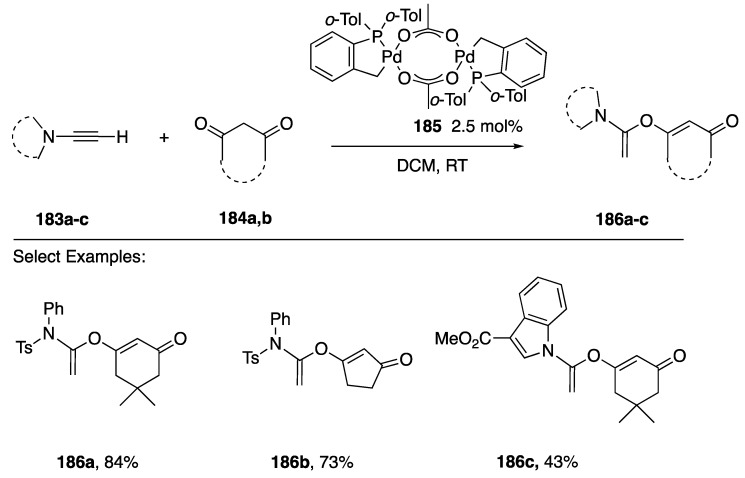
Clavier and Buono’s regioselective addition of cyclic 1,3-diones to *N*-alkynyl amides.

In 2014 Clavier and Buono reported the regioselective α-addition of 1,3-diones **184a,b** to *N*-alkynyl amides **183a,b** and the *N*-alkynyindole **183c** in the presence of the catalysis **184** to afford exclusively the products **186a**–**c** (Scheme 50) [82]. These authors note that the indole product **186c** is only formed in moderate yield.

#### 3.2.2. Carbon-Carbon Bond Formation

**Scheme 51:**
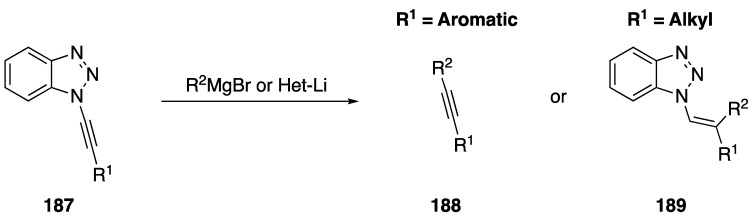
Katritzky’s synthesis of disubstituted alkynes from *N*-alkynyl benzotriazole.

The addition of carbon nucleophiles to *N*-alkynyl azoles to generate new carbon-carbon bonds has been investigated as well. In 2002, Katritzky published an addition of Grignard and organolithium reagents to *N*-alkynyl benzotriazoles **187** generating disubstituted alkynes **188** in the case of aromatic nucleophiles and *N*-vinyl benzotriazoles **189** in the case of aliphatic carbon nucleophiles (Scheme 51) [18]. In conjunction with the researchers’ method for synthesizing *N*-alkynyl benznotriazoles from acid chlorides, this addition of aromatic nucleophiles represents a unique approach to synthesizing disubstituted acetylenes from carboxylic acids.

**Scheme 52:**
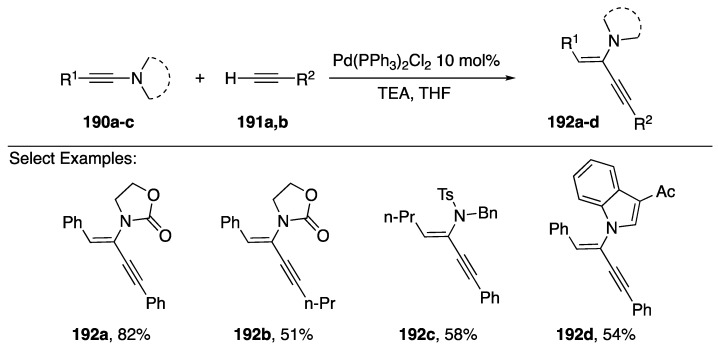
Reddy’s hydroalkynylation of *N*-alkynyl amides.

In 2015, Reddy published a palladium-catalyzed hydroalkynylation of *N*-alkynyl amides (Scheme 52) [83]. Using Pd(PPh_3_)_2_Cl_2_ as a catalyst and triethylamine as a base the researchers were able to add terminal alkynes to *N*-alkynyl amides with high levels of regio- and stereoselectivity. The reaction proceeded in good yields with many different *N*-alkynyl amide substrates **190a**–**c** including *N*-alkynyl indoles **190d**. Additionally several different terminal alkynes participated in this reaction. Interestingly, in the case of *N*-alkynyl-p-toluenesulfonamides, such as **190c**, when the substitution on the *N*-alkyne is aliphatic the hydroalkynylation proceeds with *cis*-stereoselectivity and when the substitution is aromatic the reaction proceeds with *trans*-stereoselectivity.

**Scheme 53:**
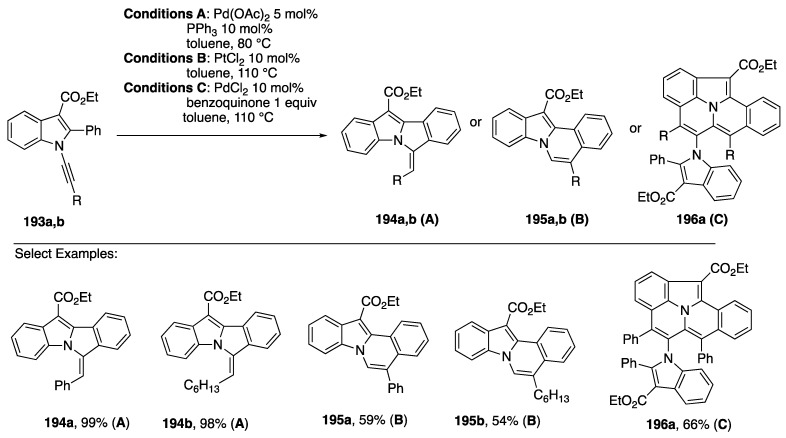
Park’s regiodivergent cyclization of *N*-alkynylindoles.

**Scheme 54:**
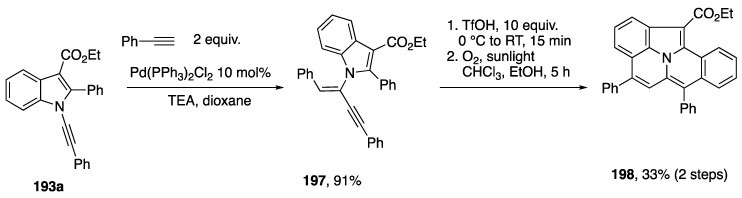
Park’s hydroalkynylation/cyclization approach to pentacyclic heterocycles.

In 2017, Park published an approach to diverse fused heterocycles based on regiodivergent cyclizations and oxidative cyclodimerization of *N*-alknylindols (Scheme 53) [84]. On one hand, *N*-alkynylindols **193** were found to undergo intramolecular hydroarylation by C-H activation with Pd(OAc)_2_ with a phosphine ligand to afford the cyclized products **194** through 5-*exo-dig* cyclization, while electrophilic addition facilitated by PtCl_2_ led to **195** through 6-*endo-dig* cyclization. Interestingly, an oxidative dimerization of **193** occurs in the presence of PdCl_2_ and an equivalent of benzoquinone to afford the pentacycles **195**. A range of aryl and alkyl substitutents on the *N*-alkynylindole **193** are tolerated in each of these cyclizations, although the yields of 6-*endo*-*dig* cyclization products **194** and dimers **195** are generally lower than the 5-*exo*-*dig* products **194.** In this same report, Park also noted that the products of hydroalkynylation (cf. Scheme 52) of *N*-alkynyl-2-phenylindole can serve as substrates for cyclization to the pentacyclic benzo[7,8]indolizino[2,3,4,5-*ija*]quinolines (Scheme 54) [84].

**Scheme 55:**
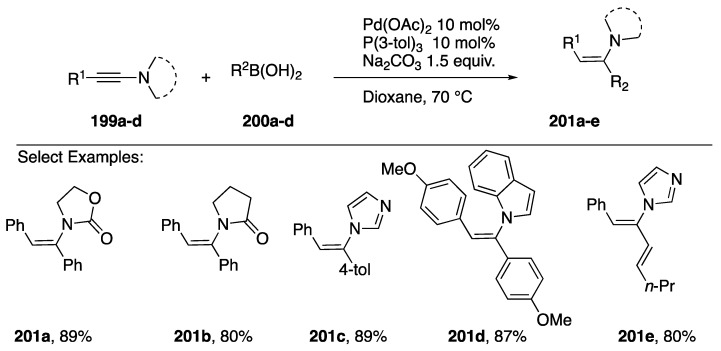
Zhu’s (*Z*)-stereoselective boronic acid coupling to *N*-alkynyl amides and azoles.

In 2014, Zhu reported a stereoselective addition of boronic acids to *N*-alkynyl amides **199a,b** and *N*-alkynyl azoles **199c,d** to afford the (*Z*)-α,β-disubstituted enamines **200a**–**e** (Scheme 55) [85]. Both aryl and alkenyl boronic acids participate in the coupling in good yields.

**Scheme 56:**
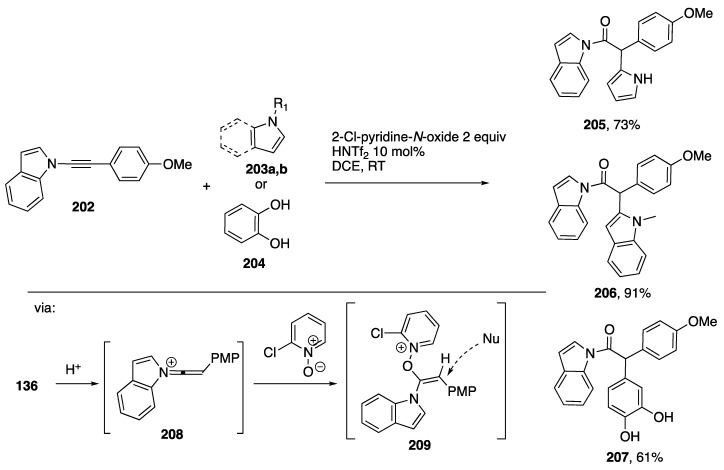
Shin’s Bronsted acid catalyzed oxygenative Friedel-Crafts coupling of an *N*-alkynlindole.

As part of an approach to access α-oxo-carbene surrogates in the absence of metal ions, Shin reported in 2017 a Bronsted acid catalyzed Friedel-Crafts-type reaction of *N*-alkynyl amides and the *N*-alkynyl indole **202** in the presence of 2-chloropyridine *N*-oxide (Scheme 56) [86]. These workers employed nucleophilic arenes such as pyrrole **203a**, indole **203b** and catechol **204** to obtain coupled products **205, 206** and **207**, respectively in good yields. In support of a proposed mechanism involving S_N2′_ displacement on the electrophilic intermediate **209**, the authors note that when reactions on *N*-alkynyl amides are carried out in the presence of a chiral dipyridine *N*,*N*’-dioxide affords coupled products with substantial enantioselectivity.

#### 3.2.3. Carbon-Boron and Carbon-Hydrogen Bond Formation

**Scheme 57:**
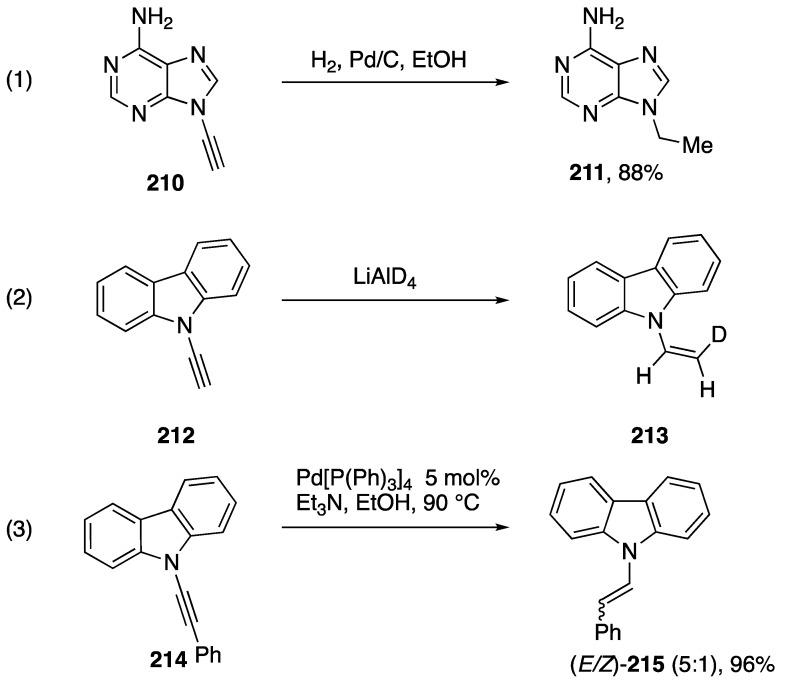
Addition of hydrogen and deuteride to *N*-alkynyl azoles.

The addition of hydrogen or deuterium to *N*-alkynyl amides and azoles is a convenient way to synthesize *N*-alkyl and *N*-vinyl azoles that may be challenging to synthesize otherwise. In 1994, Zemlicka published the reduction of *N*^9^-alkynyl adenine with hydrogen gas and palladium on carbon (Scheme 57, Reaction (1) [16]). In 2000, Bauld and Gao published the reduction of *N*-ethynyl carbazole with lithium aluminum deuteride forming *N*-(*cis*-2-deuteriovinyl)carbazole (**213**) (Scheme 57, Reaction (2) [87]). In contrast to the stereoselectivity of this transformation, Swamy reported in 2017 that the H-transfer reduction of *N*-alkynyl carbazole **214** affords a mixture of (*E*)- and (*Z*)-products **215** (Scheme 57, Reaction (3)), despite the highly (*E*)-selective reduction of *N*-alkynyl amides under these conditions [88].

**Scheme 58:**
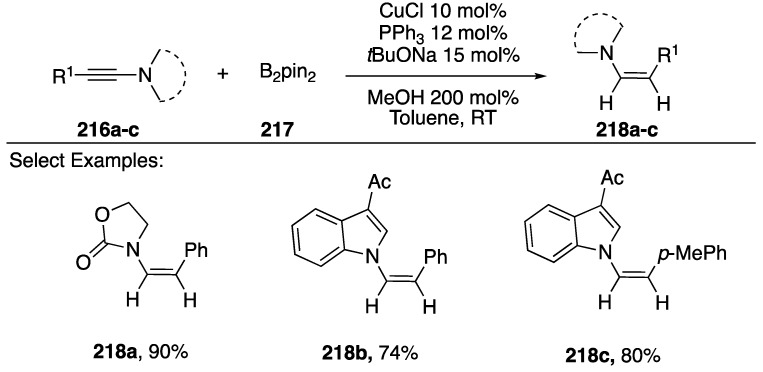
Zhu’s copper-catalyzed reduction of *N*-alkynyl amides.

In 2013, Zhu reported a copper-catalyzed reduction of *N*-alkynyl amides/indoles to *N*-alkenyl amides via a regio- and stereoselective boron addition and subsequent protonolysis (Scheme 58) [89]. The researchers found CuCl with PPh_3_ and NaO*t*Bu to be the ideal conditions to carry out this reaction. A variety of *N*-alkynyl substrates were tolerated including *N*-alkynyl oxazolidin-2-one **218a**, γ-lactams, and indoles **218b,c**. Upon further investigation of the reaction, Zhu proposed the following mechanism: 1) a copper-boryl complex adds to the alkyne via a *syn*-addition and α-boron regioselectivity, 2) protonolysis of the copper-carbon bond, 3) transmetalation of the vinyl-boron with copper, 4) another protonolysis of the copper-carbon bond. In its entirety, this reaction is a formal addition of H_2_ to *N*-alkynyl amides/indoles.

**Scheme 59:**
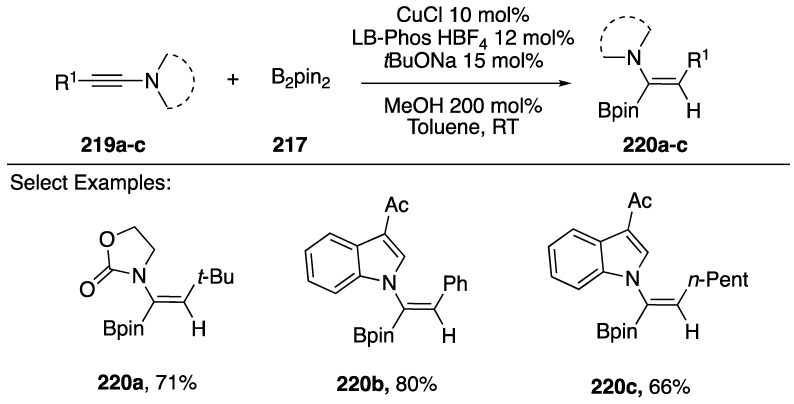
Zhu’s stereoselective hydroboration of *N*-alkynyl amides and indoles.

Hydroboration of alkynes represents an important strategy for synthesizing vinylboronates, important building blocks in the synthesis of complex organic molecules as well as intermediates for alkene preparation by protonolysis [90]. In 2014, Zhu reported a copper-catalyzed hydroboration of *N*-alkynyl amides/indoles (Scheme 59) [91]. The researchers found that slightly modifying their previous conditions for the reduction of *N*-alkynyl amides/indoles (Scheme 58) by substituting PPh_3_ with Xantphos or LB-Phos-HBF_4_ changed the selectivity of the reaction from reducing the alkyne to generating *N*-alkenyl-vinylboronates in good yields. This reaction worked well with *N*-alkynyl oxazolidin-2-ones (**220a**) and with *N*-alkynyl indoles **220b,c**.

### 3.3. Cycloadditions and Annulations

The ability of *N*-alkynyl azoles to participate in a wide variety of cycloadditionas and annulations provides ready access to a variety of fused heteroaromatic ring systems. While many of these reaction types are shared with *N*-alkynyl amides and other alkynes, there are notable instances where the course of these cyclization is unique in the case of the *N*-alkynyl azoles.

#### 3.3.1. [2 + 1] Cycloaddition

**Scheme 60:**
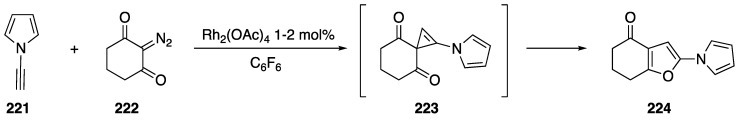
Pirrung’s [2 + 1] cycloaddition with *N*-ethynyl pyrrole.

Pirrung reported a [2 + 1] cycloaddition of a rhodium carbenoid to *N*-alkynyl pyrrole **221** in 1994 (Scheme 60) [92]. The researchers carried out the reactions with Rh_2_(OAc)_4_ as the catalyst in hexafluorobenzene. Following formation of a rhodium carbenoid with cyclic diazodiketone **222**, the carbene adds to the *N*-alkyne generating the short-lived cyclopropene **223**; subsequent rearrangement of **223** gave tetrahydrobenzofuran **224** in moderate yield. Other terminal alkynes also afforded the tetrahydrobenzofuran products in similar yields.

**Scheme 61:**
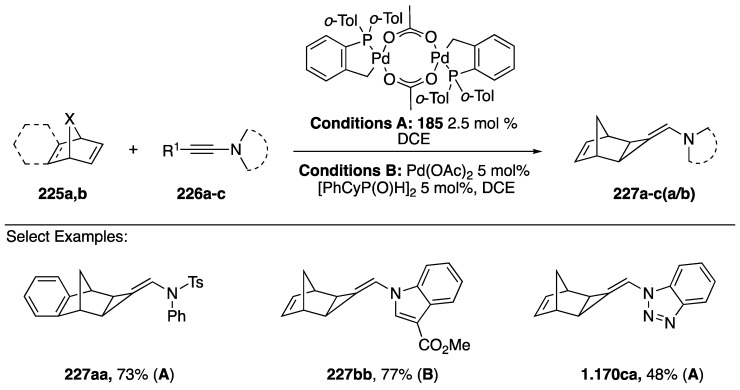
Clavier and Buono’s [2 + 1] cycloadditions of *N*-alkynyl amides/azoles.

In 2013, Clavier and Buono published a [2 + 1] cycloaddition of *N*-alkynyl amides and azoles with norbornene (Scheme 61) [93]. The researchers were interested in developing a new method for synthesizing aminomethylenecyclopropanes, a structure found in powerful antiviral agents, but with limited methods for its synthesis [94]. Ultimately Clavier and Buono found two sets of conditions (**A/B**) that successfully added *N*-alkynyl amides/azoles to norbornene. The palladium (II) catalyst used for conditions **A** had been previously known to promote the hydroalkynylation of alkynes to norbornadienes (Scheme 50). Interestingly, the researchers found that at lower reaction temperatures the same catalyst promoted the [2 + 1] addition of *N*-substituted alkynes to norbornadienes. These conditions worked well for many different norbornadienes **225a,b** and substituted alkynes **226a**–**c** to give aminomethylenecyclopropanes **227aa** and **227ca** in good yields (73% and 48%, respectively). Clavier and Buono had to use an alternative set of conditions (**B**) for the *N*-alkynyl indole substrate they tested, because the preferred catalyst gave an inseparable mixture of compounds.

#### 3.3.2. [2 + 2] Cycloaddition

**Scheme 62:**
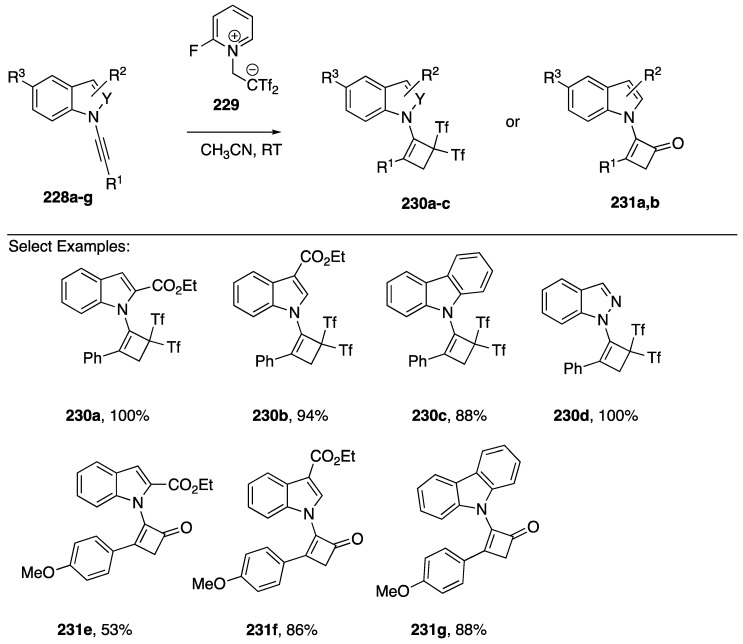
Alcaide’s Ficini cycloaddition of *N*-alkynylindoles, -carbazoles, and indazoles.

The ability of *N*-alkynyl amines and amides to undergo dipolar [2 + 2] cycloadditions with electron-deficient alkenes (Ficini reaction) is well established [1,95]. However, the application of the Ficini reaction with *N*-alkynyl azoles had not been reported until 2017 when Alcaide showed that a wide range of heterosubstituted alkynes, including *N*-alkynidoles, *N*-alkyncarbazoles, and *N*-alkynyindazole **228a**–**g** undergo Ficini reaction with 1,1-bis(trifluoromethylsulfonyl)ethane, generated in situ from the zwiterionic precursor **229** to afford the cyclobutenes **230a**–**d** or cyclobutanones **231e**–**g**, the later formed only in the case of **228e**–**g** bearing an electron-donating aryl substituent (Scheme 62) [96].

#### 3.3.3. [3 + 2] Cycloaddition

**Scheme 63:**
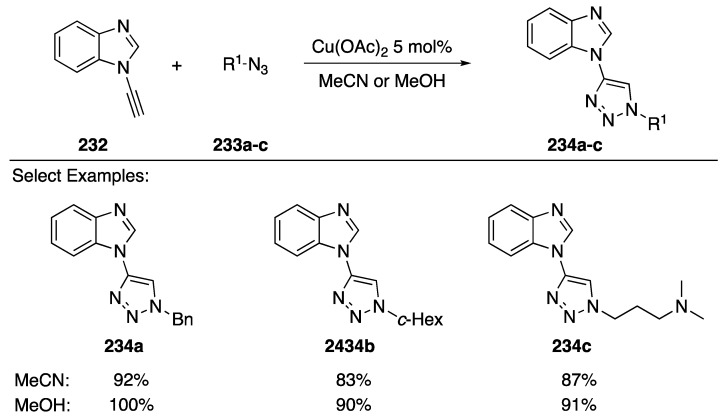
Watson and Burley’s click reaction with *N*-alkynyl benzimidazole.

In 2016, Watson and Burley disclosed a [3 + 2] copper-catalyzed “click” cycloaddition of *N*-alkynyl benzimidazole with assorted azides (Scheme 63) [97]. The researchers found that *N*-alkynyl benzimidazole **232** reacted with azides **233a**–**c**, undergoing reaction in both MeCN and MeOH solvents to afford the triazoles **234a**–**c** in excellent yields (83–100%). Interestingly, aliphatic alkynes gave no desired product when the cycloadditions were carried out in MeOH, and the researchers were able to exploit this characteristic to perform sequential chemoselective click reactions.

**Scheme 64:**
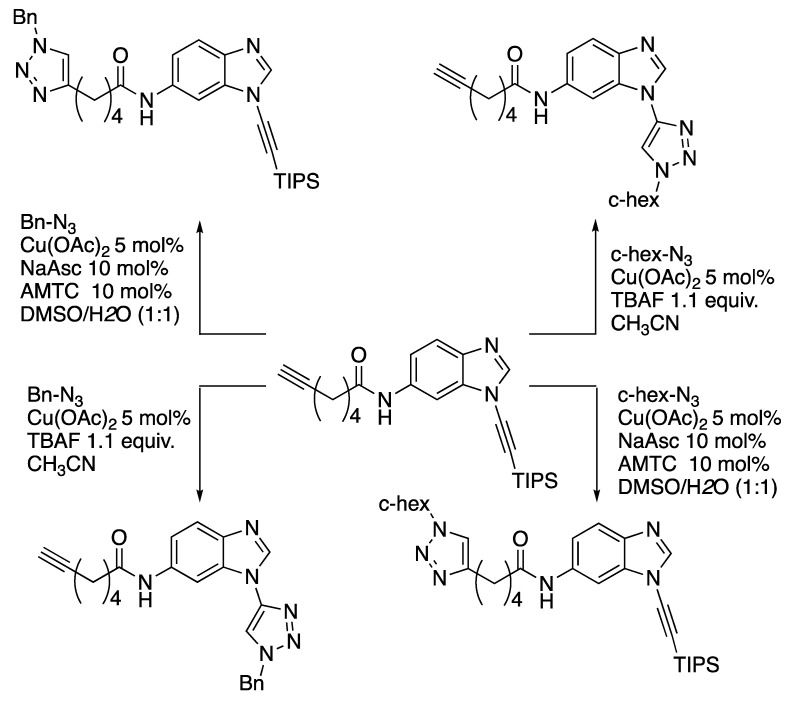
Watson and Burley’s orthogonal click-cycloaddition scheme.

Subsequent work by Burley and Watson revealed that there is a switch in the rate-determining step for triazole formation from copper-acetylide formation to azide-ligation in the case of **232** [98]. With this insight, these workers have developed an orthogonal scheme for click-cycloadditions based on tri(isopropyl)silyl-protected *N*-alkynylbenzimidazoles (Scheme 64) [99].

**Scheme 65:**
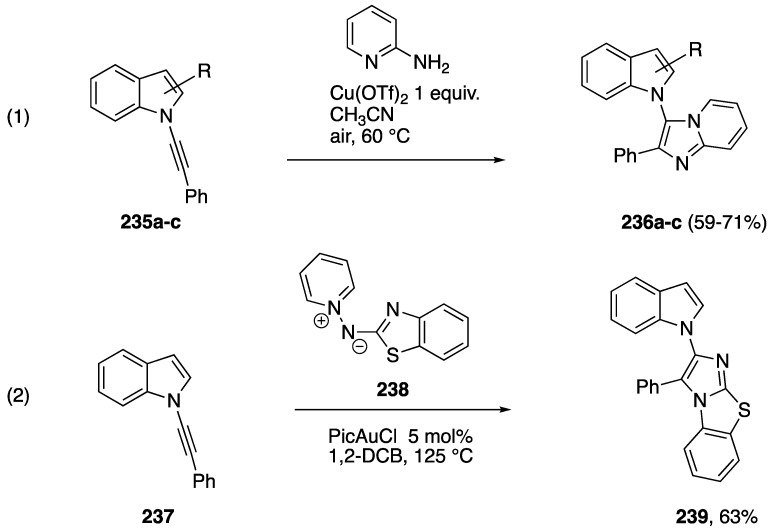
Metal-catalyzed formal [3 + 2] additions of *N*-alkynylindoles.

A number of studies have reported metal-catalyzed formal [3 + 2] additions of *N*-alkynyl amides and have included *N*-alkynyl azole examples. Reddy reported a copper-mediated addition of 2-aminopyridine to *N*-alkynyl amides and the *N*-alkynylindoles **235a**–**c** to afford the indole-substituted imidazolopyridines **236a**–**c** (Scheme 65, Reaction (1) [100]). In 2017, building up their work on gold-catalyzed formal [3 + 2] cycloadditions [101,102], Davies group reported the gold-catalyzed addition of the *N*,*N*-dipole **238** to the *N*-alkynylindole **237** to afford the fused heterocycle **239** (Scheme 65, Reaction (2) [103]).

**Scheme 66:**
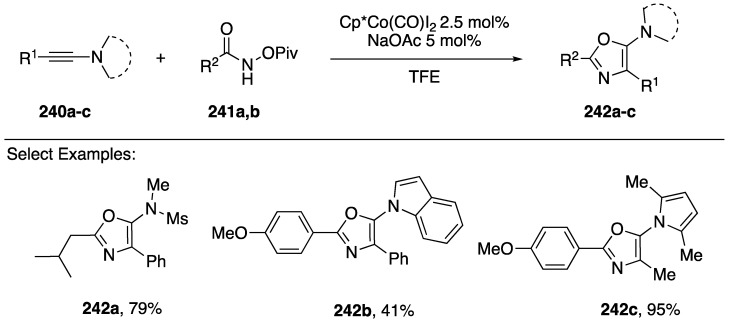
Li’s cobalt-catalyzed synthesis of 5-azole-oxazoles.

In 2017, Li published a cobalt-catalyzed synthesis of 5-aminooxazoles via a [3 + 2] cycloaddition of *N*-(pivaloyloxy)amides with *N*-alkynyl amides/azoles (Scheme 66) [104]. The researchers were interested in developing a Co(III)-catalyzed synthesis of amino-substituted isoquinolones via carbon-hydrogen (C-H) activation and were surprised when they isolated 5-aminooxazole as the sole product. Interestingly, the reaction gave the opposite regioisomer compared to previously reported metal-catalyzed syntheses of aminooxazoles [102]. 

Li found that under their optimized conditions (Scheme 66) they could synthesize a large variety of 5-aminooxazoles **242a**–**c**; the reaction tolerated both aromatic and aliphatic substitutions in the 2-position, as well as both aromatic and aliphatic substitutions in the 4-position. However, in the case of terminal *N*-alkynyl amides, the reaction gave significantly reduced yields. The researchers even performed the reaction using an *N*-alkynyl indole **242b** and an *N*-alkynyl pyrrole **242c** giving the corresponding 5-azole-oxazole products in 41% and 95% yields, respectively.

**Scheme 67:**
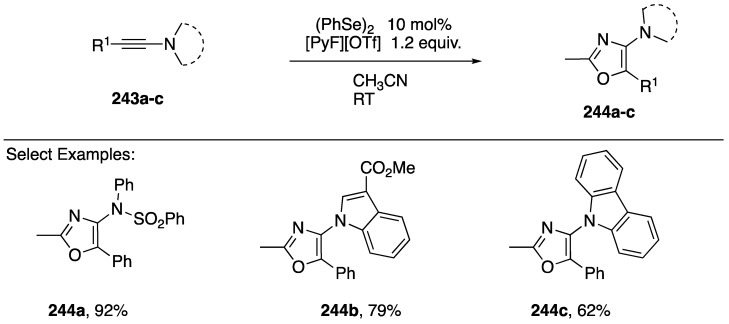
Zhou’s selenium π-acid catalyzed synthesis of 2-methylozazoles.

An alternative formal [3 + 2] addition to afford oxazoles was reported in 2018 by Zhou and co-workers (Scheme 67) [105]. They showed that a variety of *N*-alkynyl amides and *N*-alkynyl azoles **234a**–**c** undergo diphenyl diselenide-catalyzed oxidative addition of solvent acetonitrile to afford 2-methyloxazoles **244a**–**c** (Scheme 67). This process was proposed to proceed through a selenirenium ion intermediate that is trapped by acetonitrile and then undergoes oxidation and cyclization to the oxazole.

#### 3.3.4. [4 + 2] Cycloaddition

**Scheme 68:**
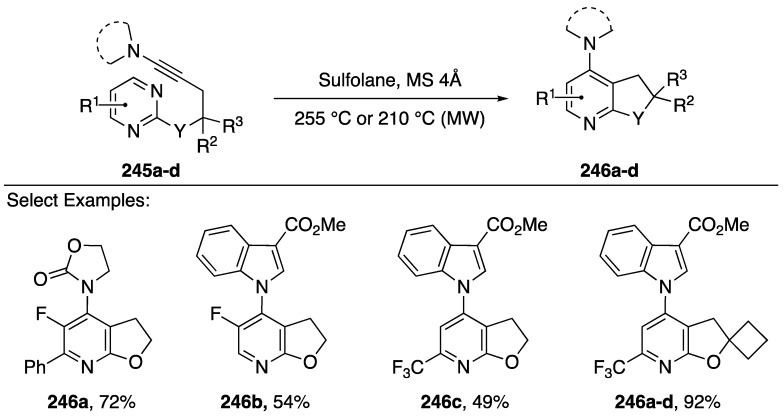
Gandon and Blanchard’s [4 + 2] cycloaddition of *N*-substituted alkynes.

In 2016, Gandon and Blanchard published an intramolecular [4 + 2] inverse electron-demand cycloaddition of *N*-alkynyl amides and indoles as a new method to generate unique fused pyridine bicycles (Scheme 68) [106]. Although the participation of *N*-substituted alkynes in cycloaddition reactions is well documented, prior to their report the reaction with pyrimidine was unknown [107]. Pyrimidines are poorly reactive as electron-deficient azadienes in [4 + 2] reactions [108], however, the researchers hypothesized that when linked with *N*-substituted alkynes the reaction pair should be adequately reactive. 

Gandon and Blanchard found that heating the starting materials **245a**–**d** by microwave irradiation in sulfolane afforded corresponding pyridines **246a**–**d** in moderate to excellent yields (49–92%). The reaction tolerated many different nitrogen-substitutions on the alkyne, including oxazolidin-2-one **246a** and 3-substituted indoles **246b**–**d**. Additionally, many different substitutions were tolerated on the pyrimidine, including phenyl (compound **246a**) fluorine (compounds **246a,b**) and trifuoromethyl (compounds **246c,d**). Following their initial report, in 2017, Gandon and Blanchard published a full paper that includes a DFT study of this inverse electron demand Diels-Alder/retro-Diels-Alder sequence [109].

#### 3.3.5. [2 + 2 + 2] Cycloaddition

**Scheme 69:**
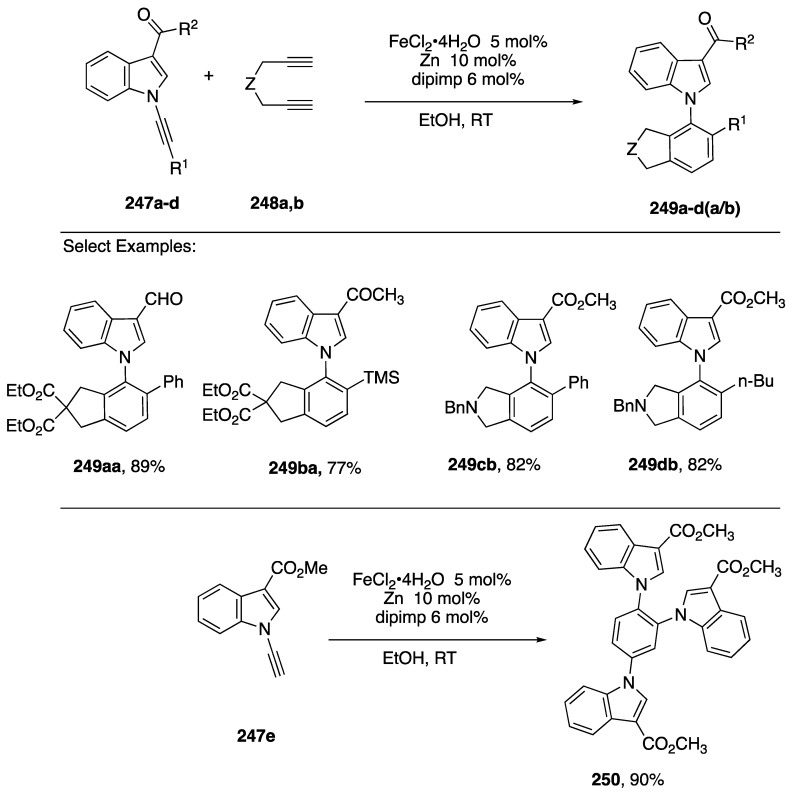
Goswami’s [2 + 2 + 2] route to *N*-arylindoles and tri-indolylbenzenes.

In 2015, Goswami published a study of a metal-catalyzed [2 + 2 + 2] to *N*-arylindoles from *N*-alkynylindoles [110]. After screening a number of metal catalysts, they found that *N*-alkynylindoles **247a**–**c** reacted with the diynes **248a,b** in the presence of catalytic FeCl_2_·4H_2_O, zinc, and ligand (*E*)-*N*-(2,6-diisopropylphenyl)-1-(pyridin-2-yl)methanimine (dipimp) in ethanol to afford the *N*-arylindoles **249a**–**d(a/b)** (Scheme 69). 

Although only 3-carbonylindole partners **247a**–**d** were explored, a wide range of alkyne substitutents R^1^ were tolerated. While the reaction proceeded well with symmetrical, terminal diynes **248a,b**, attempts to carry out the reaction with 1,7-octadiyne failed and reactions with non-diterminal diynes afforded mixtures of regioisomeric products. Similarly, attempts to carry out a [2 + 2 + 2] trimerization of **247a** afforded a regioisomeric mixture of tri-indole-substituted benzene products; however, trimerization of the *N*-ethynylindole **247e** gave a single product **250** in excellent yield.

#### 3.3.6. [3 + 2 + 2] Cycloaddition

**Scheme 70:**
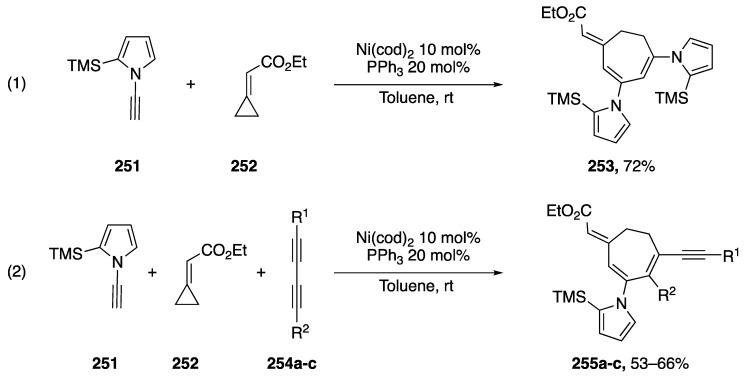
Saito’s [3 + 2 + 2] cycloaddition with *N*-alkynyl pyrrole.

Saito reported a nickel-catalyzed [3 + 2 + 2] cycloaddition of ethyl-cyclopropylideneacetate and *N*-alkynyl pyrrole in 2010 (Scheme 70) [111]. The researchers were interested in the use of heteroatom-substituted alkynes as substrates in three-component cycloadditions to construct complex cycloheptadienes in one step. Saito started his investigations with *O*-substituted alkynes, but also documented the [3 + 2 + 2] cycloaddition of *N*-substituted alkynes. In their investigations, the researchers found that *N*-alkynyl carbamates reacted with ethylcyclopropylidene acetate (**252**) to give mixtures of [3 + 2 + 2] and cyclotrimerization products. However, Saito reported that in the case of *N*-ethynyl pyrrole substrate **251**, the reaction with **251** proceeded smoothly giving only the desired cycloaddition product **252** in a 72% yield (Scheme 70, Reaction (1)). The researchers also reported a cycloaddition using diacetylenes **253a**–**c** as substrates in the [3 + 2 + 2] reaction with *N*-ethynyl pyrrole **251** (Scheme 70, Reaction (2)). Due to differences in sterics and electronics Saito was able to isolate the desired products, **253a**–**c**, in moderate yields.

#### 3.3.7. [4 + 3 + 2] Cycloaddition

**Scheme 71:**
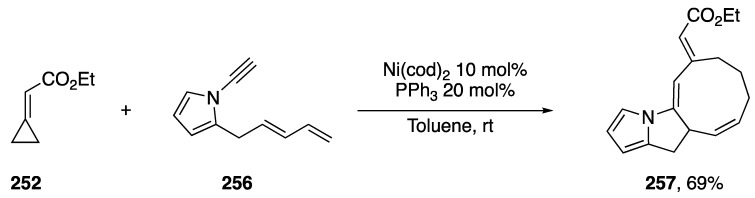
Saito’s nickel-catalyzed [4 + 3 + 2] cycloaddition with *N*-alkynyl pyrrole.

In 2013, Saito published a nickel-catalyzed [4 + 3 + 2] cycloaddition of ethyl-cyclopropylideneacetate **252** with a number of dienynes (Scheme 71) [112]. The researchers thoroughly investigated the scope of dienyne substrates, which included the *N*-alkynyl pyrrole dienyne **256**. Under Saito’s optimized conditions, dienyne **256** reacted to give the polycyclic heterocycle **257** in a 69% yield. Their method represents just one of a just a few transition metal-mediated synthesis of 9-membered carbocycles.

#### 3.3.8. Other Annulations

**Scheme 72:**
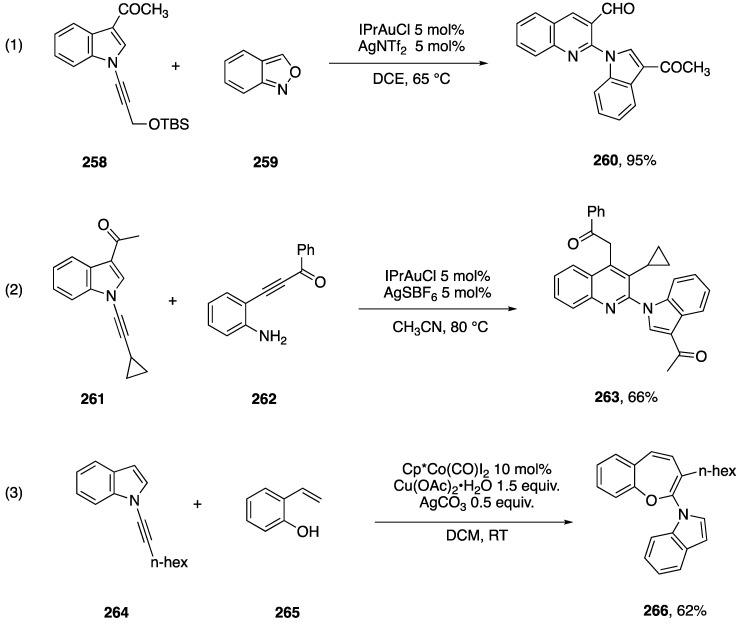
Other annulations of *N*-alkynylindoles.

There are a few examples of other annulation reactions involving *N*-alkynylindoles that have been reported during studies focused on *N*-alkynyl amide annulations. Xie and Hashmi reported in 2016 a gold-catalyzed preparation of quinolines from propargyl ethers and benzo[*c*]isoxazole **259** (Scheme 72, Reaction (1) [113]). 

In the course of examining the scope of propargyl ethers that participate in this reaction, they reported that *N*-alkynylindole **258** affords excellent yield of the quinolone **260.** In 2018, Arcadi and co-workers reported an alternative route to quinolones via a gold-catalyzed annulation of *N*-alkynyl amides with β-(2-aminiphenyl)-α,β-ynones [114]. During this work they found that a range of *N*-substituted alkynes, including the *N*-alkynylindole **261** adds to the ynone **262** affording the quinoline **263** (Scheme 72, Reaction (2)). In 2018, Li and co-workers reported an oxidative cobalt-catalyzed [2 + 5] addition of ortho-vinylphenols with *N*-alkynyl amides [115]. In exploring the scope of this annulation they found that the *N*-alkynylindole **264** cleanly affords the benzoxepine **266** (Scheme 72, Reaction (3)). In contrast, the corresponding *N*-phenylethynylindole afforded mixtures of regioisomers.

### 3.4. Bergman Cyclizations

**Scheme 73:**
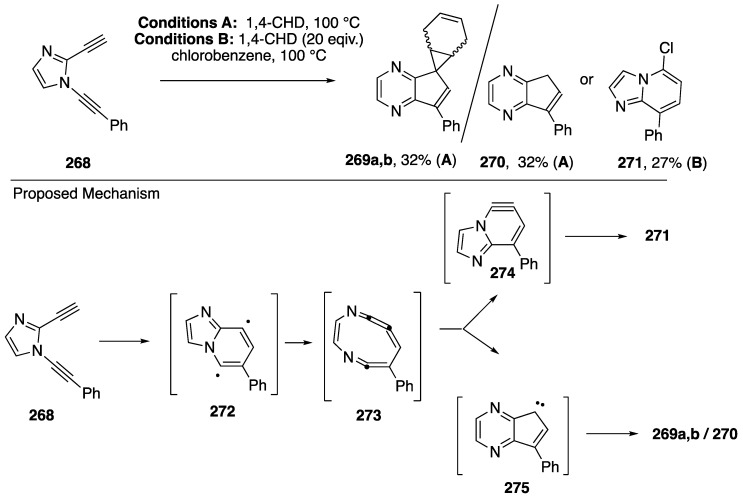
Kerwin’s aza-Bergman cyclization-based rearrangements of dialkynylimidazoles.

The Bergman cyclization has garnered the attention of many chemists due to the naturally occurring enediyne cores of many natural products that can generate diradicals via the Bergman cyclization and cleave DNA, imparting potent cytotoxicity to these compounds. As part of a research program aiming to temper the cytotoxicity and improve on the lack of selectivity of Bergman diradicals towards DNA cleavage, Kerwin reported in 2002 the surprising results from the thermolysis of the heterocyclic 3-aza-3-ene-1,5-diyne **268** (Scheme 73) [31]. The *N*-alkynyl imidazole **268**, when heated in neat 1,4-cyclohexadiene (1,4-CDH) afforded the cyclopentapyrazines **269a,b** as a mixture of stereoisomers together with the cyclopentapyrazine **270**. In contrast, when the thermolysis was carried out in chlorobenzene containing 1,4-CHD, the imidazolopyridine **271** was isolated. Based upon these results and a subsequent study demonstrating the generality of these transformations [32], it was proposed that these products are the result of an unprecedented aza-Bergman/retro-aza-Bergman cascade involving initial formation of the diradical **272** which undergoes collapse to the cyclic cumulene **273**. Cyclization of **273** to the carbene **275** followed by trapping with 1,4-CHD leads to the products **269a,b** and **270**, while an alternative cyclization to the benzyne **274** and trapping by solvent leads to **271.**

**Scheme 74:**
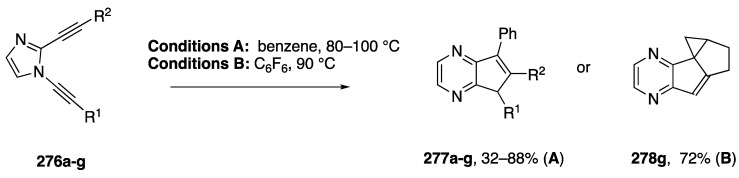
Kerwin’s dialkynylimidazole route to cyclopentapyrazines.

In 2006, Kerwin reported the synthetic application of these dialkynylimidazole rearrangements (Scheme 74) [116]. Thermolysis of the dialkynylimidazoles **276a**–**g** in benzene affords moderate to good yields of the benzene-trapped products **277a**–**g**. In the case of a dialkynylimidazole with a pendant alkene, thermolysis in hexafluorobenzene instead affords the tetracyclic product **278g**. Additional work showed that the benzyne intermediate (cf, **274**, Scheme 73) can also be intercepted with HCl to afford chloroimidazolopyridine products [117].

### 3.5. Sigmatropic Rearrangements

**Scheme 75:**
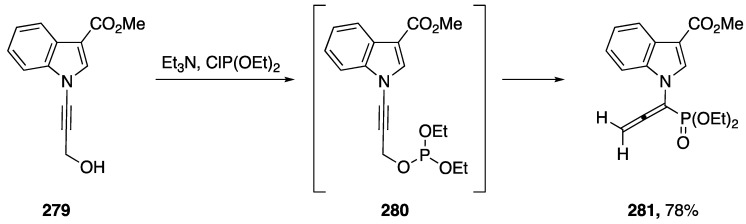
Rabasso’s [2,3]-sigmatropic rearrangement of *N*-alkynyl indole.

In 2012, Rabasso published a [2,3]-sigmatropic rearrangement of *N*-alkynyl amides [118]. The following year, 2013, during their investigations into the selective reduction of amino-allenephosphonates, Rabasso expanded the scope of their [2,3]-sigmatropic rearrangement to include *N*-alkynyl indole (Scheme 75) [119]. The researchers found that compound **279**, underwent a [2,3]-rearrangement when treated with diethyl chlorophosphite, giving *N*-allenylphosphate **281** in a 78% yield. Unfortunately, Rabasso was unable to achieve a selective reduction with **281**.

**Scheme 76:**
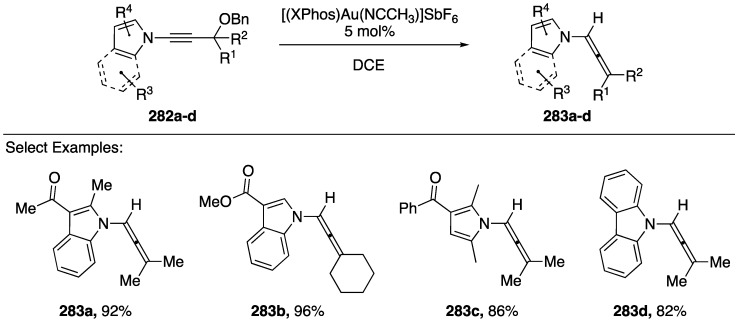
Zhao and Gagosz’s gold-catalyzed hydride shift of *N*-alkynyl azoles.

In 2017, Zhao and Gagosz published a gold-catalyzed hydride shift towards the synthesis of *N*-allenyl amides/azoles from *N*-alkynyl amides/azoles (Scheme 76) [120]. Under optimized conditions, the researchers found that a large number of *N*-alkynyl amides and azoles were tolerated in this reaction (**282a**–**d**): *N*-alkynyl indole, pyrrole, and carbazole all gave the respective allene products (**283a**–**d**), additionally a variety of R^1^ and R^2^ substitutions were tolerated. Zhao and Gagosz also discuss a cascade reaction with *N*-alkynyl indoles and pyrroles in which the initially formed allene reacts with the electron-rich heterocycles forming new 5-membered rings (not shown).

### 3.6. Other Reactions

**Scheme 77:**
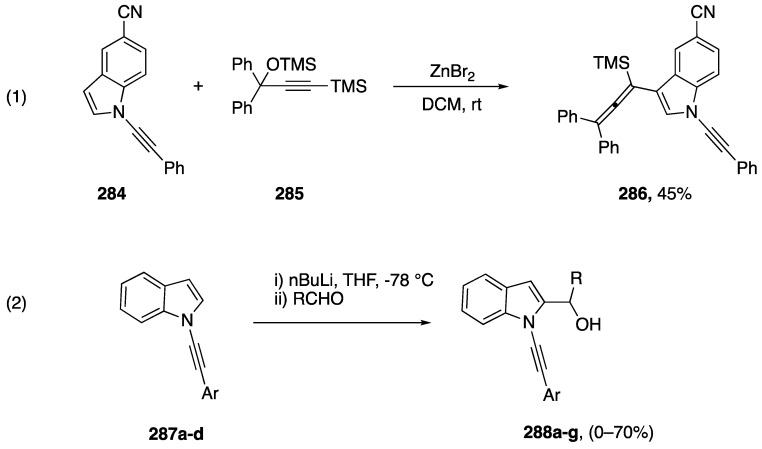
Functionalization of *N*-alkynyl indoles.

In addition to the transformations discussed above, which focus on reactions at the alkyne of *N*-alkynyl azoles, there are numerous examples of reactions in which the *N*-alkynyl group is inert, allowing further functionalization of the azole core. Many of these are incorporated into the above section as routes to the various *N*-alkynyl azole substrates. In addition, there have been a number of reports the focus on these functionalization reactions. While investigating the ZnBr_2_ catalyzed reaction of *N*-alkynyl amides with benzylic alcohols Cao and Xu reported an unexpected reaction of *N*-alkynyl indole (Scheme 77, equation 1) [121]. While other *N*-substituted alkynes reacted under these conditions in a nucleophilic fashion to the benzylic alcohols, *N*-alkynyl indole **284** was inert, instead reacting at the 3-position with substrate **285** generating *N*-alkynyl indole **286** in a 45% yield. In 2018, Zeni reported the 2-functionalization of *N*-alkynyl indoles **287a**–**f** by deprotonation with *n*-butyl lithium followed by trapping with a variety of aldehydes to afford the 2-substituted compounds **288** (Scheme 77, equation 2) [122]. No products were obtained from *N*-alkynyl indoles **287** bearing *p*-methoxyphenyl or *o*-chlorophenyl substituents, and the scope of aldehydes is generally limited to substituted benzaldehydes or formaldehyde; although, alternative electrophiles such as benzophenone and chlorosilanes also afforded products.

## 4. Applications of *N*-Alkynyl Azoles

The explosion of interest in the synthesis of *N*-alkynyl azoles over the last 15 years has fueled a burgeoning interest in the exploration of various applications of these compounds. *N*-alkynyl azoles have found applications in the total synthesis of natural products, the synthesis of polymers, and they have had their physical and biological properties evaluated.

### 4.1. Total Syntheses

**Scheme 78:**
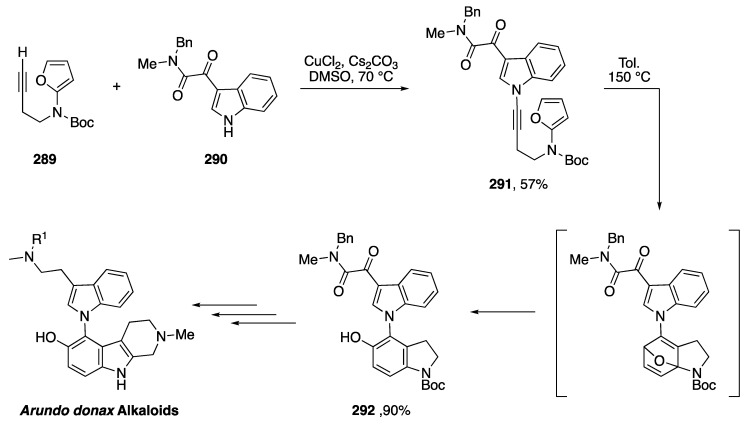
Beaudry’s synthesis of the bis-indole alkaloids from *Arundo donax.*

In 2014, Beaudry published an *N*-alkynyl indole Diels-Alder strategy for the synthesis of the bis-indole alkaloids from *Arundo donax* (Scheme 78) [123]. Beaudry’s strategy involved the initial synthesis of *N*-alkynyl indole **291** from alkyne **290** and indole **289**. Using conditions inspired by Stahl, *N*-alkynyl indole **291** was isolated in a 57% yield. A subsequent Diels-Alder reaction at 150 °C efficiently produced **292** in a 90% yield, completing the core of the natural products. Over several more steps the researchers were able to complete the synthesis of arundamine, arundanine, arundacine, and arundarine. Interestingly, Beaudry was able to show that these structures form atropisomers that could be resolved with a chiral HPLC. The researchers determined the half-lives of racemization to be between 1 and 7 h.

**Scheme 79:**
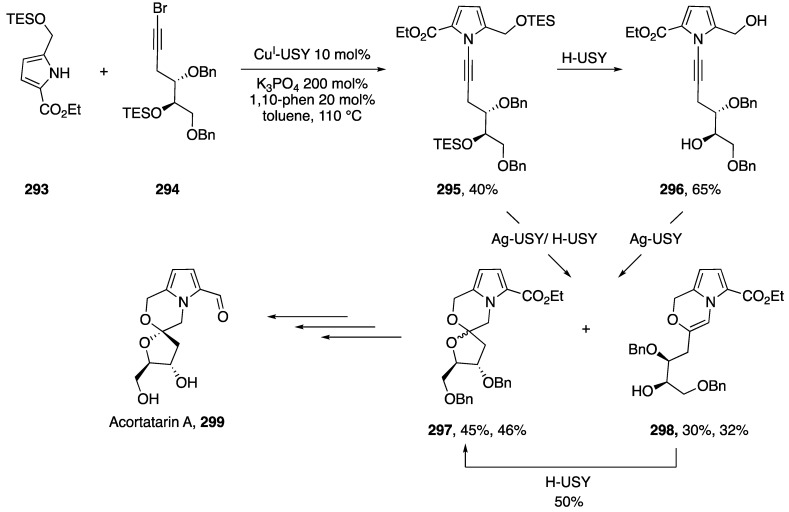
Pale and Beneteau’s zeolite strategy towards acortatarin A.

In 2017, Pale and Beneteau published a zeolite based organic synthesis of acortatarin A using an *N*-alkynyl pyrrole retrosynthetic strategy (Scheme 79) [124]. 

The researchers used zeolite catalysis for the key steps of their synthesis. The first instance being a Cu^I^-USY catalyzed cross-coupling of 1-bromoalkyne **294** and pyrrole **293** to give *N*-alkynyl pyrrole **295** in a 40% yield. A subsequent spiroketalization of **295** was carried out with Ag-USY and H-USY to give spiroketal **297** in a 45% yield and the partially cyclized product **298** in a 30% yield. Pale and Beneteau found that separating the steps and first forming diol **296** with H-USY and then carrying out the spiroketalization did not help improve the yields. Additionally, the researchers were able to finish the spirocyclization of **298** using a H-USY zeolite. Following a few finishing steps, acortatarin A was synthesized with a 3:1 dr.

### 4.2. Polymer Synthesis

**Scheme 80:**
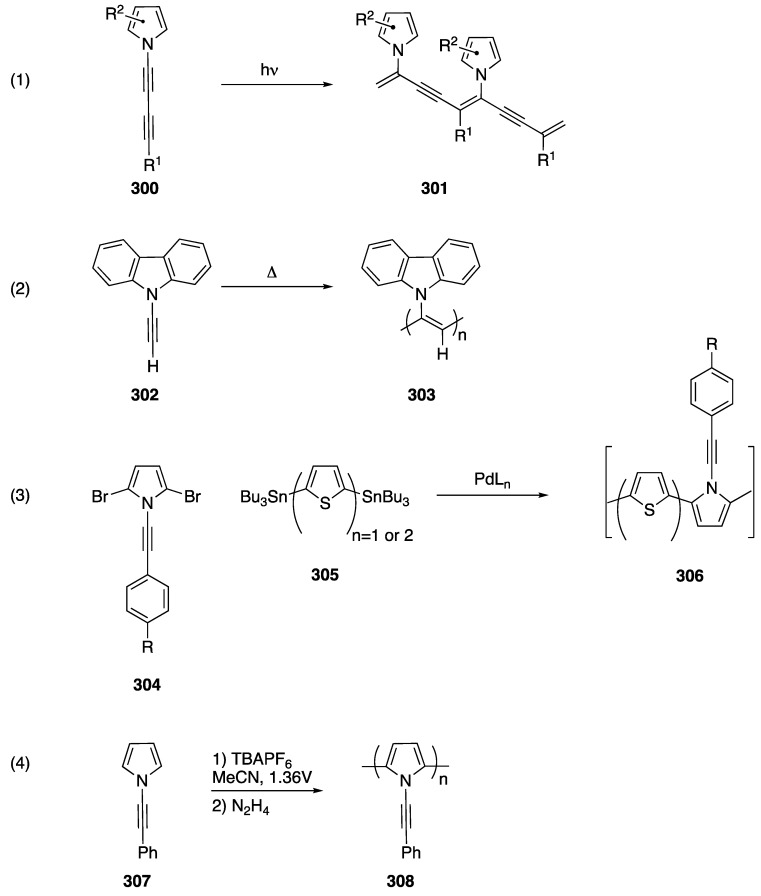
Application of *N*-alkynyl azoles in polymerization reactions.

Some of the earliest reports of *N*-alkynyl azoles in the literature involve their synthesis and subsequent polymerization. One of the more studied examples is the polymerization of the diacetylenes of *N*-alkynyl pyrrole and carbazole **300** (Scheme 80, Reaction (1) [14,60,125]). 

The diacetylene polymers **301** were often studied for their optical properties. Polyacetylene polymers of *N*-alkynyl azoles have been investigated as well. In 1970, Okamoto published the synthesis of poly-*N*-ethynyl carbazole **303** and studied the conductivity of the polymer (Scheme 80, Reaction (2) [8]). Subsequent studies on this and related polymers have appeared [125,126,127]. Additionally, polymerizations in which the alkyne remains intact have been reported. Yamamoto published the co-polymerization of *N*-alkynyl pyrroles **304a,b** with the di(stanyl)thiophenes **305a,b** (Scheme 80, Reaction (3) [128,129,130]). Yamamoto also reported the oxidative polymerization of **307** with TBAPF_6_ in 2012 (Scheme 80, Reaction (4) [131]). Okamoto also published a paper in 1973 investigating the photoconductive properties of arylethynylcopper polymers including the copper polymer of *N*-ethynyl carbazole [132].

### 4.3. Other Applications

*N*-alkynyl azoles have been studied for their physical properties. In 1998, Dellepiane published a paper investigating the vibrational properties of several diacetylenes, including a diacetylene of *N*-alkynyl carbazole [133]. Additionally, in 2013, Sambri, Tonelli, and Armaroli studied donor-acceptor luminophores made from *N*-alkynyl carbazole [134]. Trolez has reported on the optoelectronic properties of *N*-alkynylcarbazole- and indole derivatives [135]. Burley has studied the metal ion coordination of triazoles derived from *N*-alkynyl benzimidazoles [136].

In addition to studies of their physical properties, there have been several reports investigating the biological properties of *N*-alkynyl azoles. Zemlicka disclosed their investigations into the biological activity of *N*^9^-alkynyl adenine in 1994 [16]. They found *N*^9^-alkynyl adenine to be a substrate of moderate activity for adenosine deaminase, in addition it inhibited the growth of murine leukemia L1210, and suppressed growth of mouse and human colon tumors C38, H8 or H116. In 2009, Kerwin published a paper disclosing their biological evaluation of dialkynylimidazoles specifically designed to target p38α kinase [137]. Using this scaffold, these workers developed a molecular probe that led to the discovery of a novel class of p-38α docking-recognition site inhibitors [138]. In future studies, Kerwin investigated the ability of dialkynylimidazoles to induce apoptosis in A549 cancer cells, and the researchers also investigated whether aza-Bergman rearrangement rates could be used to predict cytotoxicity [64]. Bhattacharjee has also investigated the cytotoxicity of *N*-alkynyl pyrazoles [56].

## 5. Conclusions

This review has covered the synthesis and reactions of *N*-alkynyl azoles, a small subclass of nitrogen-substituted alkynes, which have received a lot of attention over the last several decades. Initially researchers synthesized these molecules using classical dehydrohalogenation chemistry that limited the scope of accessible substrates; however, with the discovery of milder catalytic protocols for the synthesis of *N*-alkynyl amides and azoles the synthesis of more complicated structures containing sensitive functional groups has become possible. Additionally, these catalytic protocols have brought more attention to the reactions of *N*-alkynyl azoles and the various potential applications of the compounds. *N*-alkynyl azoles have been used in metal-catalyzed addition reactions, they have found tremendous use in cycloaddition reactions, they have been applied to the total synthesis of several natural products, and have been investigated for their biological activity and use in polymers.

We believe the importance of both azoles and alkynes to the organic community will ensure a continued interest in *N*-alkynyl azoles. Notably, although there has been significant improvement in the procedures for their synthesis, we believe that continued efforts towards developing even more efficient methods would be of value to the synthetic community. Additionally, further work still needs to be done towards understanding the distinct reactivity of the many different *N*-alkynyl azoles. On a final note, due to the importance of azoles within the pharmaceutical industry we believe the application of *N*-alkynyl azoles for the synthesis of natural products and designed molecules will continue to grow in the future.
